# Advancing Carbon Nanotube Fibers: Addressing Challenges from Production to Application

**DOI:** 10.1021/acscentsci.5c00155

**Published:** 2025-06-03

**Authors:** Hao Guo, Chunlei Dong, Qingquan Han, Hongyu Jiang, Hongji Sun, Xuemei Sun, Songlin Zhang, Huisheng Peng

**Affiliations:** State Key Laboratory of Molecular Engineering of Polymers, Department of Macromolecular Science, Institute of Fiber Materials and Devices, and Laboratory of Advanced Materials, 12478 Fudan University, Shanghai, 200438, China

## Abstract

Carbon nanotube (CNT) fibers, renowned for their theoretically high tensile strength, low density, and outstanding electrical conductivity, are promising candidates for cutting-edge applications in wearable electronics, bioengineering, and aerospace engineering. Despite their immense potential, the widespread adoption of CNT fibers faces critical barriers, including the challenge of enhancing macroscopic fiber performance and achieving scalable, consistent production. The extraordinary intrinsic properties of individual CNTs are not fully transferred to macroscopic fibers due to weak intertube interactions, misalignment, and structural defects. Among the available production methods, floating catalyst chemical vapor deposition stands out for its promise to enable the large-scale synthesis of CNT fibers. However, achieving consistent quality and scalability via this technique remains a significant obstacle. This outlook highlights the importance of innovative strategies for multiscale performance optimization and advances in scalable fabrication methodologies. A distinctive perspective on CNT fiber production is provided, emphasizing the integration of machine learning with process optimization strategies to enhance the uniformity and efficiency. The outlook systematically discusses these challenges, exploring strategies for multiscale performance optimization, scaled-up fabrication methodologies, and efficient manufacturing processes. Additionally, it examines the transformative applications of CNT fibers across diverse industries, underscoring their potential to revolutionize next-generation technologies.

## Graphical abstract

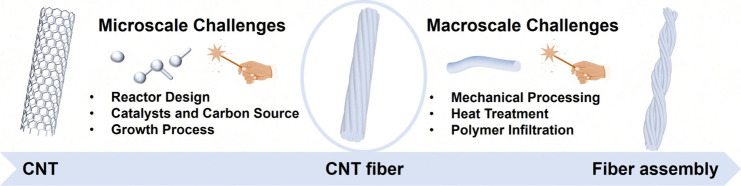

## 1 Introduction

Carbon nanotube (CNT) fibers have attracted significant attention in recent years because of their extraordinary mechanical, electrical, and thermal properties, which make them highly versatile for a wide range of advanced applications.^1–4^ These fibers exhibit remarkable mechanical characteristics, including a tensile strength that is much greater than that of steel, high thermal conductivity, low thermal expansion, and exceptional electrical conductivity. Owing to their high strength- or conductivity-to-weight ratio, CNT fibers are ideally suited for lightweight structural components in aerospace applications.^5–7^ Furthermore, their outstanding electrical conductivity and thermal stability make them prime candidates as key materials for flexible electronics, energy storage devices, sensors, and thermal management systems.^8–10^ However, realizing the full potential of CNT fibers necessitates addressing critical challenges related to performance advancement, batch-to-batch material consistency, and scalable production scalability.

Compared to conventional carbon and graphene fibers, CNT fibers uniquely integrate high electrical conductivity, mechanical robustness, and flexibility.^11,12^ While carbon fibers are widely utilized in aerospace and automotive industries, their electrical conductivity remains relatively low.^13^ Graphene fibers, on the other hand, exhibit exceptional conductivity but face challenges in mechanical performance and large-scale production.^14,15^ CNT fibers offer a well-balanced combination of strength, conductivity, and lightweight properties, positioning them as promising candidates for multifunctional applications, such as structural electronics and smart textiles.^16^ However, the practical realization of these advantages hinges on the development of scalable fabrication techniques that enable precise control over the CNT alignment, purity, and structural integrity. Advancing these production methods is therefore crucial to fully unlocking the potential of CNT fibers.

The fabrication process of CNT fibers plays a decisive role in determining their performance, consistency, and scalability. Achieving high-quality CNT fibers requires overcoming key challenges, such as optimizing the alignment of individual CNTs, enhancing the structural integrity of CNT bundles, and minimizing structural defects during the chemical synthesis process. Among various techniques, wet spinning offers simplicity and scalability but struggles with issues such as insufficient mechanical strength in the resulting fibers.^17,18^ In contrast, array spinning improves individual CNT alignment but is limited by stringent processing conditions for scaleup fabrication.^19^ Floating catalyst chemical vapor deposition (FCCVD) has emerged as a leading production method for directly spinning CNT fibers by shrinking the CNT aerogel sock. By supplying the carbon sources and transition metal catalysts into the tube furnace, FCCVD enables continuous and potentially largescale production of CNT fibers.^20^ This method streamlines the fabrication process of CNT fibers and significantly enhances their yield.^21^ The comparative table evaluates various fabrication methods as Table 1. Despite the potential scalability, the FCCVD approach is still confronted with challenges, including catalyst uniformity, structural defects, and property consistency, which limit its broader industrial application. Recent advancements have been achieved by advancing the FCCVD reactor design such as optimizing the tube geometry and controlling the temperature gradient and airflow rate. Consequently, carbon deposition uniformity is improved with a better control on the structural defects and alignment of individual CNTs, potentially facilitating the scalability of high-quality CNT fibers.^22–24^

**Table 1:** Comparison of Various Fabrication Methods^a^

**Method**	**Uniformity**	**Tensile strength**	**Electrical conductivity**	**Production scalability**	**High cost-efficiency**	**Environmentally friendly**
Wet spinning	★	★	★	★★	★	★
Array spinning	★★★	★★★	★★★	★	★	★★
FCCVD	★★	★★★	★★★	★★★	★★★	★★★

a Note: A higher number of stars indicates an advantageous effect on the respective category.

In this outlook, we examined the challenges faced by CNT fibers at both the microscale and macroscale levels and presented potential solutions. We also discussed two critical issues in continuous production of CNT fibers via FCCVD: continuity and uniformity (Figure 1).^25^ Finally, promising applications of CNT fibers across various fields were showcased. By surmounting these multiscale structural challenges and enhancing production scalability, CNT fibers are poised to achieve widespread adoption and drive advancements in next-generation technologies.

**Figure 1:**
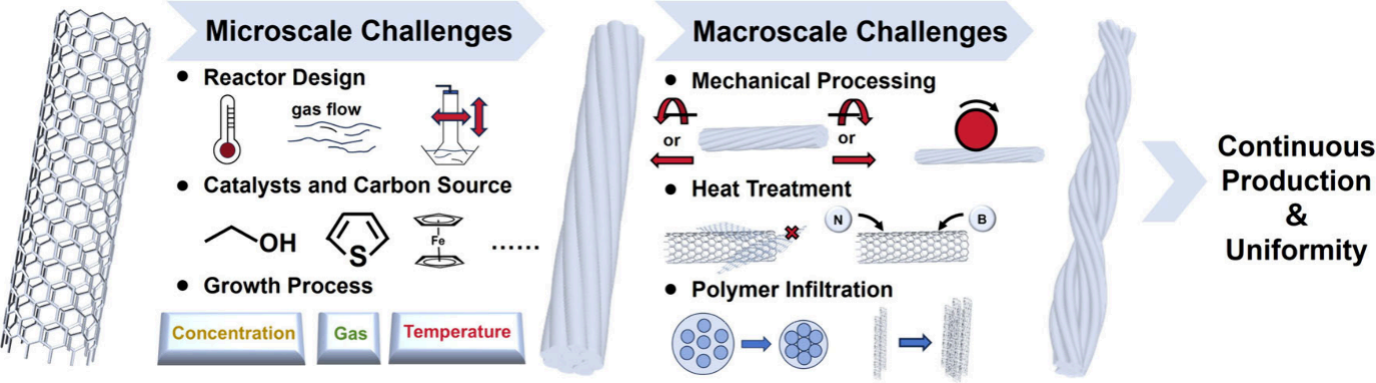
Challenges and potential solutions for CNT fibers at the microscale and macroscale levels.

## 2 Microscale Challenges: Unlocking the Potential of Individual CNTs

Despite the intrinsically high tensile strength and excellent electrical conductivity of individual CNTs, noncovalent interactions between neighboring CNTs within CNT fibers significantly hinder efficient intertube load or electron transfer. Additionally, microscale structural defects within CNT fibers prevent them from fully leveraging the exceptional properties of individual CNTs. These limitations are further exacerbated by nanoscale complexities, including misalignments of CNTs or CNT bundles and atomic structural defects. Addressing these challenges requires optimizing the FCCVD reactor designs, refining carbon and catalyst precursor materials and implementing innovative processing strategies.

### 2.1 Improvement in Reactor Design

The synthesis of individual CNTs in FCCVD furnaces consists of three primary stages: high-temperature decomposition of carbon sources, catalytic growth of CNTs, and CNT aerogel sock deposition to eventually form the fiber. Effective thermal gradient management is essential for optimizing these processes. Uniform heating across the reactor length ensured consistent catalytic reactions of the precursor materials. Enhancements by incorporating ceramic heating rods have been shown to reduce thermal gradients, therefore minimizing amorphous carbon formation and increasing carbon conversion rates to as high as 90%.^28^ It significantly improves aspect ratios of individual CNTs—up to a 50% increase, thereby laying a strong foundation for scalable production of high-quality CNT fibers.

The flow regulation of carrier gas is another critical factor which is closely related to the geometry of the tube furnace, determining flow patterns and growth outcomes. Vertical reactors enhance laminar flow while minimizing turbulence and ensuring uniform temperature distribution, which enables consistent CNT growth and efficient collection (Figure 2a). In contrast, horizontal reactors are more prone to vortex formation, which can lead to particle retention and catalyst deactivation.^23^ Tailored reactor designs, such as narrowing the reactor diameter to increase the gas flow velocity, further optimize the flow dynamics. Higher velocities suppress vortex effects, reduce catalyst aggregation, and minimize secondary reactions and impurities, thereby increasing the CNT fiber uniformity in terms of physical properties.^29^

**Figure 2:**
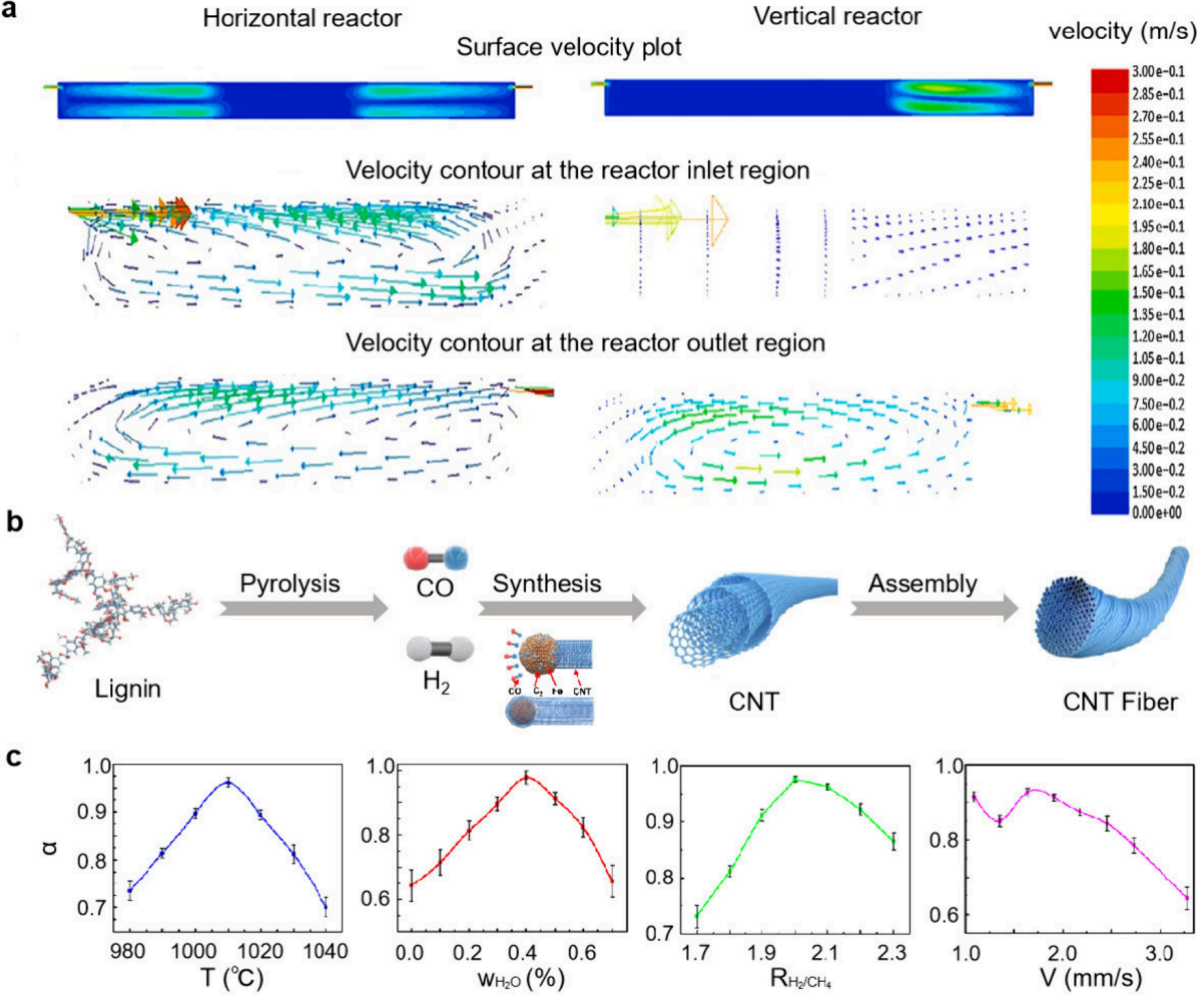
Solutions to the microscale challenges. (a) Computational fluid dynamics simulation results of the velocity vector profile and velocity contours at reactor inlet and outlet regions. The horizontal reactors are more prone to vortex formation. Adapted with permission from ref.^25^ Copyright 2013 John Wiley and Sons. (b) Synthesis mechanism of CNT fibers from lignin and the growth mechanism on iron catalysts. Adapted from ref.^26^ Available under a CC-BY (Creative Commons Attribution 4.0 International License) license. Copyright 2022 F. Liu et al. (c) The relation between catalyst activity probability (α) and growth temperature, water content, H_2_/CH_4_ and gas velocity. Adapted from ref.^27^ Copyright 2013 American Chemical Society.

However, while reducing turbulence and controlling temperature gradients have been effective in small-scale experiments, maintaining these parameters in large-scale FCCVD reactors remains a major challenge. The complex gas dynamics in industrial reactors can lead to inconsistent CNT growth, negatively impacting fiber uniformity and mechanical performance. To address this, integrating computational fluid dynamics (CFD) simulations and real-time process monitoring into the reactor design is essential to ensure stable production conditions. These strategies help optimize flow dynamics, reduce turbulence-induced inconsistencies, and improve CNT fiber uniformity on an industrial scale.

Recent studies underscore the advantages of spatial confinement in FCCVD systems. Narrowing the furnace geometry enhances the trapping of catalyst nanoparticles and carbon atoms, facilitating uniform catalyst deposition and a high-density CNT arrangement. This controlled environment ensures consistent catalytic activities and uniform nucleation sites, enabling the synthesis of CNT fibers with improved structural and electronic properties.^30^ Spatial confinement also minimizes the recirculation of unreacted carbon precursors and suppresses vortex-induced disturbances, thereby increasing the reaction efficiency and scalability.

### 2.2 Optimization of Catalysts and Carbon Sources

Catalyst performance is a pivotal factor in determining the growth rate, yield, and quality of the CNTs. Recent studies in catalyst design, exemplified by advanced bimetallic systems and nanoengineered supports integrated into continuous synthesis processes, have enabled unique control over catalyst particle size and distribution, thereby markedly accelerating CNT growth rates and enhancing uniformity.^31^ Thus, the choice of catalyst precursors plays a crucial role in tuning the reaction dynamics and optimizing the CNT synthesis.

Functionalized ferrocene derivatives are particularly promising catalyst precursors. For example, the hydroxyl group (−OH) in ferrocene methanol reduces amorphous carbon deposition and mitigates surface oxidation, thereby extending the catalyst lifespan and maintaining high activity.^32^ This leads to more controlled CNT growth, faster growth rates, improved crystallinity, and minimized structural defects. Conversely, acetylferrocene, which contains a carbonyl group (C=O), enhances the reaction activity but may increase carbon contamination under certain conditions, compromising the uniformity and yield.^31^ Moreover, recent optimizations using metal–organic frameworks (MOFs) such as ZIF-67 have further refined continuous synthesis processes by ensuring a steady supply of high-surface-area cobalt nanoparticles, thus bolstering both growth kinetics and structural uniformity of the CNTs.^22^ Upon decomposition, ZIF-67 releases cobalt nanoparticles with high surface areas and thermal stability, providing a steady supply of active catalytic sites for CNT growth. This approach has been shown to produce single-walled CNTs (SWCNTs) at rates of up to 159.39 mg/h, significantly improving structural uniformity, reducing the amount of amorphous carbon, and narrowing the diameter distributions, all of which are crucial for high-performance CNT fibers.

Equally critical is the choice of the carbon source. For instance, the selection of a multicomponent carbon source, such as a mixture of ethanol and thiophene, has been demonstrated to significantly influence the growth of CNTs in FCCVD. Ethanol serves as a clean, efficient carbon source, while thiophene, with its sulfur content, acts as a promoter, enhancing the growth process and facilitating uniform CNT formation.^33^ Additionally, biomass-derived materials, such as lignin and tannic acid, offer promising alternatives to petroleum-based sources. During lignin pyrolysis, carbon monoxide and hydrogen—key components for CNT growth—are released, enabling production rates of up to 120 m/h with excellent mechanical and electrical properties. Optimizing the ratio of carbon monoxide to hydrogen during pyrolysis can further enhance CNT growth while reducing structural defects (Figure 2b).^26^ Similarly, tannic acid serves as both a carbon source and a reducing agent, ensuring the stability of catalyst active sites and promoting uniform carbon atom deposition, thereby minimizing defects and enabling uniform CNT growth.^34^ Biomass-derived carbon sources hold significant potential for industrial applications. As a renewable resource, they can be obtained from agricultural and forestry waste, thereby reducing production costs.^35^ Additionally, their surfaces are rich in functional groups and heteroatoms, which may enhance the performance of CNT fibers.^36^ Therefore, biomass carbon sources are expected to play a vital role in the future industrial production of CNT fibers.

Although multicomponent carbon sources have been shown to enhance CNT growth, their industrial application must also consider factors such as cost, stability, and byproduct generation. Similarly, although MOF-derived catalysts demonstrate high efficiency, concerns persist regarding their large-scale synthesis and long-term stability. Future research should focus on developing cost-effective, high-stability catalysts and optimizing continuous catalyst feeding strategies to maintain consistent CNT growth in industrial production.

### 2.3 Regulating the Growth Process

Innovative strategies have been developed to optimize the CNT growth mechanisms. One study introduced a “substrate interception and direction strategy” (SIDS) in a modified FCCVD process to synthesize ultralong CNTs.^37^ A flat substrate was used to intercept and reorient short floating CNTs via van der Waals interactions at the substrate edges. Computational fluid dynamics simulations revealed that the substrate induced localized turbulence, immobilizing the CNT tips while allowing their free ends to float in the gas flow. This “flying kite” tip-growth mechanism extended the growth duration and improved CNT alignment under laminar flow. Compared with conventional methods, the resulting ultralong CNT structures achieved an areal density exceeding 6700 CNTs/mm^2^—an improvement of 2–3 orders of magnitude. Raman spectroscopy confirmed minimal structural defects, demonstrating the effectiveness of the FCCVD-SIDS integration.

Another study utilized the Schulz–Flory distribution to model the CNT length distribution and systematically optimized the catalyst activity parameters.^27^ By adjustment of factors such as the growth temperature, water concentration, and gas ratio (e.g., H_2_:CH_4_), researchers have increased the catalyst activity probability (R) to 0.995, corresponding to a catalyst deactivation probability of just 0.005 per millimeter of growth (Figure 2c). These optimized conditions enabled the synthesis of CNTs up to 550 mm in length, establishing a new benchmark for both length and structural integrity. A furnace-moving technique was employed to maintain consistent thermal zones, further extending the achievable CNT length. This work highlights the critical role of precise control over catalyst activity and gas flow parameters in reducing structural defects and achieving high-quality ultralong CNTs at scale.

Due to the numerous variables involved in the CNT production process, traditional trial-and-error experimental methods are insufficient for optimizing parameters. Recent studies have introduced machine learning (ML) techniques to navigate the multidimensional parameter space, identify optimal synthesis conditions, and facilitate the development of CNTs with specific properties. Multiparameter optimization models establish correlations between synthesis parameters (e.g., carbon feed, temperature, and catalyst amount) and CNT properties, enabling predictive control over fiber quality, yield, and scalability. However, the application of ML in FCCVD-based CNT synthesis remains limited. Most ML models are designed for the CVD method used in CNT array growth, which shares some similarities with FCCVD. ML algorithms—including artificial neural networks (ANN), random forests (RF), support vector machines (SVM), eXtreme gradient boosting (XGBoost), and Bayesian optimization (BO)—have demonstrated high accuracy in modeling CNT array growth.^38–41^

Furthermore, ML has been shown to significantly improve experimental efficiency and shorten research timelines. For instance, dynamic optimization via BO accelerated experimental speed by more than five times.^42^ When combined with automation, over 500 experiments and millions of virtual simulations were completed within just 43 days, achieving the highest precision in producing HACNT arrays at a specified density.^43^ From an economic perspective, integrating ML into industrial-scale CNT production offers promising advantages. ML-driven optimization can help unlock the complex growth mechanisms in FCCVD, bridge the performance gap between experimental and theoretical values, and reduce production costs and time compared with traditional methods. These advancements are expected to accelerate the industrialization of CNT fibers, making scalable manufacturing more economically viable.

Laboratory studies typically optimize individual variables under controlled conditions, whereas industrial production must account for environmental temperature fluctuations, reactor aging, and raw material inconsistencies. These factors introduce significant challenges in maintaining consistent CNT growth rates and fiber quality. To bridge this gap, machine-learning-driven adaptive control systems can dynamically adjust production parameters in response to real-time process data. While significant progress has been made in research settings, translating these advancements to industrial-scale production requires further refinement. Future efforts should refine ML-based optimization techniques and explore alternative reactor configurations to enhance the CNT fiber quality and production rates.

Based on our production experience and relevant studies, Table 2 summarizes key parameters influencing CNT growth in the FCCVD process along with their impact on fiber quality and corresponding optimization strategies.

**Table 2:** Properties of CNT Fibers at Different Growth Conditions

**Growth Parameter**	**Key Characteristics and Significance**	**Effects on CNT Fiber Properties**	**Optimization Strategies**
Growth Temperature (°C)	1000–1300 °C (Typical FCCVD range)	Low yield, structural defects (low T); catalyst deactivation, amorphous carbon (high T)^26,44^	High-temperature-stable catalysts (MOF-derived); thermal field optimization
Gas Flow Rate (sccm)	500–5000 sccm (Depending on reactor size and CNT production needs)	Catalyst aggregation, misalignment (low flow); turbulence, fiber irregularity (high flow)^29,45^	CFD simulations; reactor design optimization
Catalyst Type	Fe, Co, Ni, MOF-derived catalysts	CNT diameter, crystallinity, length variation^46,47^	MOF-derived catalysts; defect reduction
Carbon Source	Ethanol, toluene, methane, biomass	CNT yield, tube diameter, defect density variation^23,48^	Multicomponent carbon sources; yield enhancement
Reactor Configuration	Horizontal and vertical reactors	Horizontal: More High turbulence, misalignment (horizontal); improved uniformity (vertical)^23,49^	Vertical reactor design; turbulence minimization

## 3 Macroscale Challenges: Enhancing Mechanical Properties of CNT Fibers

At the macroscale, translating the exceptional intrinsic properties of individual CNTs into application-ready fibers remains a formidable challenge. Key obstacles include weak van der Waals forces between CNT bundles and their inferior alignments within CNT fibers. Low packing density of CNTs in CNT fibers often exhibits voids that reduce the contact area between CNT bundles, significantly weakening their mechanical and electrical properties. Addressing these issues requires optimizing the fiber structure and post-treatment techniques to minimize voids, increase the packing density, and establish a superior alignment of the CNTs.

### 3.1 Mechanical Processing

Mechanical processing can significantly improve the overall properties of CNT fibers via enhancing the CNT alignment, packing density, and void elimination. Techniques such as mechanical dual-drawing methods have shown substantial enhancement in terms of achieving better alignment of CNT bundles. A high drawing ratio (>20%) stretches CNT bundles along the fiber axis, thus reducing interstitial gaps or voids. Consequently, a high packing density of CNTs within CNT fibers can be obtained, boosting the tensile strengths up to 4.6 GPa (Figure 3a).^50,56^ These structural enhancements enable more efficient load transfer and electron transmission among neighboring CNTs, resulting in significant improvements in tensile strength and electrical conductivity compared to untreated fibers. Similarly, mechanical roller pressing also facilitates the compacting of CNTs by applying an external pressure. Concomitantly, this process reduces porosity and increases the contact area among CNTs, thereby improving the stress transfer efficiency, tensile strength, and elastic modulus of the fibers. (Figure 3b).^51,57^

**Figure 3:**
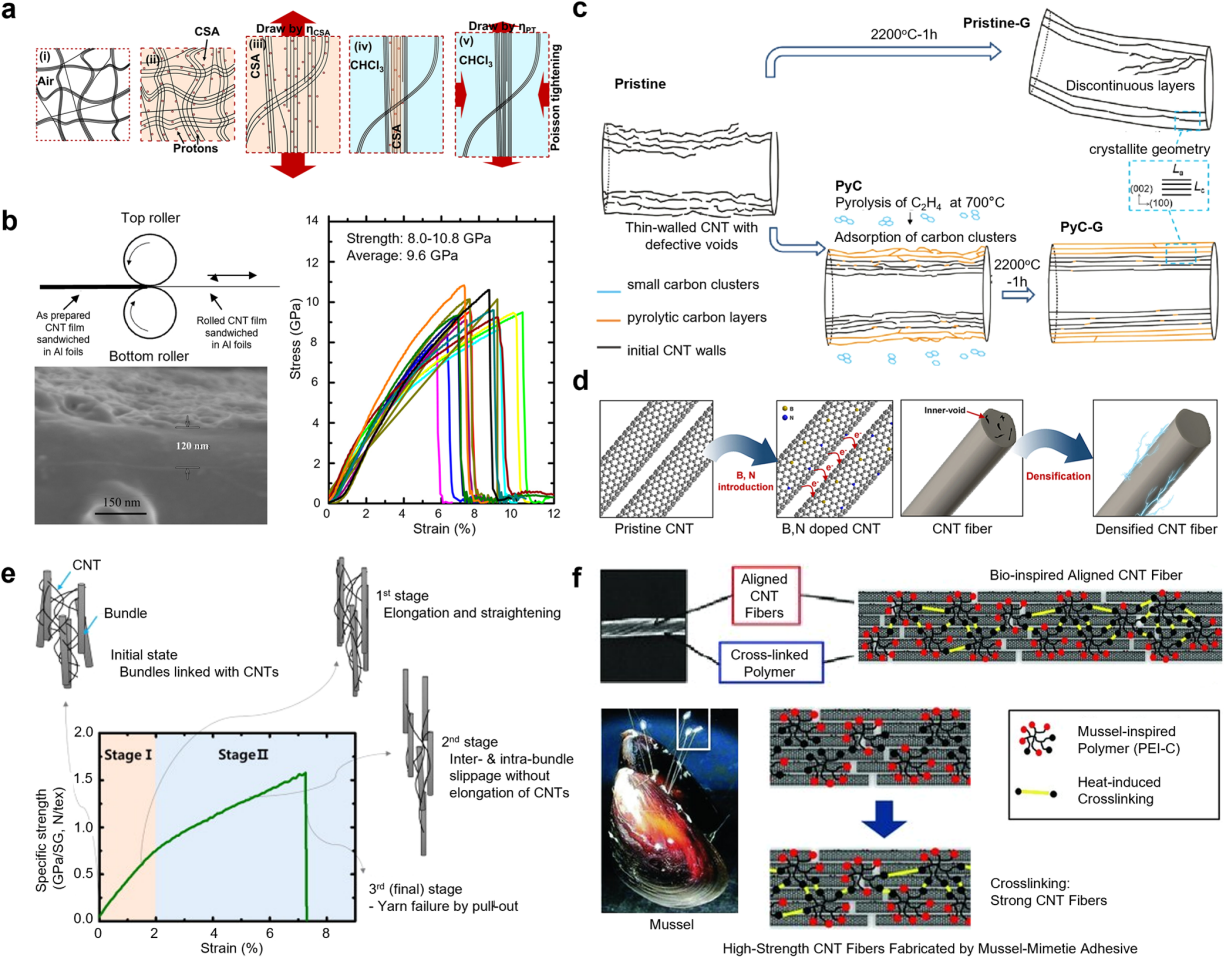
Solutions to macroscale challenges. (a) Schematic diagram of the double-drawing process used to enhance CNT fibers. The alignment and compactness are substantially enhanced via immersion in HSO_3_Cl and Poisson-effect-assisted drawing. Adapted from ref.^50^ Available under a CC-BY (Creative Commons Attribution License 4.0) license. Copyright 2022 X. Zhang et al. (b) Controlling the winding rate and applying rolling techniques improves the alignment and densification of CNTs in the film, resulting in tensile strength up to 9.6 GPa. Adapted from ref.^51^ Copyright 2016 American Chemical Society. (c) Schematic diagram of the graphitization mechanism between one-step annealed CNTs (Pristine-G) and two-step annealed CNTs (700PyC-G). Adapted with permission from ref.^52^ Copyright 2021 Springer Nature. (d) Schematic figures of boron and nitrogen doping, along with densification of CNT fibers. Adapted with permission from ref.^53^ Copyright 2021 Elsevier. (e) Deformation behavior of the polymer-infiltrated CNT fibers. Adapted with permission from ref.^54^ Copyright 2015 Elsevier. (f) Schematic illustration of mussel-inspired CNT fibers and their reinforcement performance. Adapted with permission from ref.^55^ Copyright 2011 John Wiley and Sons.

Mechanical twisting is another technique for structure optimization by improving the internal toughness and stress transfer efficiency.^58^ This mechanical process reduces voids, minimizes structural discrepancies between the interior and exterior in the fiber, and improves uniformity and strength utilization.^59,60^ However, twisting can also introduce localized stress concentrations and microscale voids due to uneven CNT distributions, which may compromise the performance of CNT fibers.^61^ To address these issues, precise control of the twisting process is essential to balancing the CNT orientation, packing density, and fiber uniformity. Recent studies indicate that combining mechanical stretching with controlled twisting significantly increases the density and reduces porosity, thus optimizing the structural integrity and mechanical performance of CNT fibers.

To further explore the impact of mechanical processing techniques on CNT fibers, Table 3 presents a comparison of four commonly used methods.

**Table 3:** Comparison of Mechanical Processing Techniques for CNT fibers

**Method**	**Effect on CNT fibers**	**Challenges**
Drawing	CNT alignment, tensile enhancement^50,56^	Structural defects
Twisting	Uniformity, strength utilization^59,60^	Potential performance reduction
Stretching	CNT alignment, tensile strength^62,63^	Uniform stretching control
Rolling	Increased packing density, strength, conductivity^57,64^	Agglomeration, flexibility reduction

### 3.2 Heat Treatment

Heat treatment is an effective strategy to repair structural defects of CNTs. For example, thermal annealing can effectively repair structural defects and improves CNT alignment and densification by reducing voids and irregularities in the microstructure.^65^ A two-step annealing process—comprising epitaxial pyrolytic carbon deposition followed by high-temperature graphitization, was reported to achieve 10% increase in tensile strength and 35% improvement in the Young’s modulus for CNT fibers via FCCVD (Figure 3c). These improvements underscore the strong correlation between enhanced microstructural uniformity and mechanical performance.^52^

Additionally, synergistic doping of CNTs with boron and nitrogen atoms can be realized during thermal treatments. As a result, electronic band structure of CNTs was significantly optimized to reduces the tunneling barrier and intertube distance (Figure 3d).^53^ This method increases the specific tensile strength to 1.2 GPa/(g·cm^–3^). It should be noted that the thermal treatment temperature is critical (e.g., 1600 °C), so as to preserve the tubular structure of CNTs. Otherwise, excessively high temperatures (e.g., 1800 °C) disrupt CNT structures with a degraded performance.

### 3.3 Modification Treatment

The structure and mechanical properties of CNT fibers can be significantly enhanced through the critical technique of polymer infiltration. Infiltrated polymers improve the inter- or intrabundle bonding strength through molecular-level coupling among CNTs.^54^ The polymer affinity determines the CNT bundle size. For example, low-affinity polymers, such as poly­(vinyl alcohol) (PVA), form larger CNT bundles that increase the contact area, thereby increasing the shear strength and load transfer efficiency (Figure 3e). The polymer coatings on CNT bundle surfaces restrict slippage, contributing to enhanced mechanical property. Compared with high-affinity polymers such as polystyrene, PVA has proven to be more effective in achieving high-density CNT packing and structural optimization, offering a practical approach to develop high-performance CNT fiber materials.

Additionally, researchers have explored bioinspired cross-linked adhesives to reinforce the CNT fibers. For example, polyethylenimine (PEI) grafted with catechol has been infiltrated into CNT fibers and cross-linked at 120 °C, resulting in a significant increase in fiber strength to 2.2 GPa (Figure 3f).^55^ The infiltration ensures uniform reinforcement by allowing the adhesive polymer to fully penetrate the internal structure of the CNT fibers. Scanning electron microscopy (SEM) and fracture analysis have revealed a shift in failure mechanisms—from sliding between individual CNTs or CNT bundles to localized fractures within CNTs, emphasizing the role of cross-linked adhesives in enhancing interfacial bonding and mechanical strength.

Furthermore, infiltrating metals (e.g., metal deposition) into CNT fibers offers another technique to substantially enhance the performance of CNT fiber composites.^66^ For instance, silver nanoparticles were uniformly deposited on the surface of CNTs to improve the wettability between CNTs and the copper matrix. The embedded silver nanoparticles significantly increase the load transfer efficiency of the fiber composite. Silver nanoparticle incorporation could repair the surface defects of CNTs. Compared with pure copper, the CNT-Ag/Cu composite results in a 32% increase in tensile strength and a 24.8% improvement in ductility. Table 4 presents a comparison of various interface engineering methods.

**Table 4:** Comparison of Various Interface Engineering Methods

**Method**	**Mechanism**	**Advantages**	**Challenges**
Covalent functionalization	Covalent bonds (e.g., C–O, C–N)^67^	Strong bonding, stable	CNT damage, complex process
π-π interactions	van der Waals forces (π-clouds)^68^	No CNT damage, simple	Weak binding, low stability
Interfacial enhanced phase	Stress dispersion in multiphase composites^69^	Multifunctionalizable	Poor compatibility, nonuniform
Hierarchical interfaces	Load transfer optimization^66,70^	Resilient, designable	Complex fabrication

Mechanical processing primarily enhances the high-density stacking of CNTs through physical methods, while modification treatments strengthen intertube connections via chemical approaches. Heat treatment improves structural order by repairing defects. These techniques collectively enhance the mechanical and electrical properties of the CNT fibers. During modification and heat treatment, additional substances or atoms may be introduced, adjusting fiber properties through mechanisms such as interfacial engineering and energy band modulation. While these secondary phases can be beneficial, they may also disrupt CNT alignment and hinder the expression of the fibers’ intrinsic properties. Therefore, multiple factors affecting the fiber properties must be carefully balanced during post-treatment.

## 4 Challenges in CNT Production: Breaking through Bottlenecks

To enable the industrial application of CNT fibers via the FCCVD method, addressing technical challenges in two critical areas—continuous production capacity and fiber material consistency—is essential. Continuous production requires effective management of carbon deposition (inactive carbon for CNT growth). While achieving uniformity of fiber properties necessitates advanced posttreatment techniques. These strategies provide vital pathways for realizing the efficient and controlled production of CNT fibers.

### 4.1 Continuous Production: Toward 10,000-m-Long CNT Fibers

Carbon deposition occurs when carbon sources and catalyst residues adhere to the inner surfaces of the furnace. Such inactive carbon inhibits catalyst activity and obstructs continuous production of CNT fibers. A few approaches have been explored to mitigate carbon deposition. For example, alkali treatments of the furnace tubes can create potassium-based protective layers (e.g., KOH) on the reactor walls, which prevent catalyst aggregation, thereby reducing carbon deposition and increasing the purity of synthesized CNTs.^22^ Additionally, interactions between the protective layer and the furnace tube can generate CO_2_ and H_2_O, favorably removing deposited amorphous carbon from catalyst surfaces and preserving their catalytic activity. Furthermore, optimizing the temperature distribution of the furnace can minimizes catalyst deactivation which guarantee the consistent growth of CNTs.^23^ The use of thermally stable carbon sources, such as methane, or mixed carbon sources with a regulated supply can further alleviate inactive carbon deposition issues, thereby supporting the stable and scalable production of CNT fibers.

The integration of machine learning (ML) into CNT fiber production represents a transformative advancement in addressing continuous production challenges. ML-driven adaptive systems enable dynamic adjustments of the production conditions to reduce the defects of individual CNTs. For example, the DeepCNT-22 machine learning force field has provided insights into defect formation and healing mechanisms at the CNT-catalyst interface, underscoring the importance of controlled synthesis conditions for achieving the growth defect-free CNTs.^24^ Moreover, combining ML with advanced characterization techniques automates data analysis, improving throughput and accuracy of CNT synthesis.^41^ Multiparameter optimization models establish correlations between synthesis parameters (e.g., carbon feed, temperature, and catalyst amount) and CNT properties, facilitating predictive control over quality, yield, and scalability of CNT fibers.^40^

### 4.2 Uniformity: Expanding the Application of CNT Fibers

Achieving property consistency of CNT fibers is critical for expanding their potential applications across diverse fields. Ethanol, as a key post-treatment chemical, plays a multifaceted role in improving fiber uniformity.^71^ First, ethanol effectively removes surface impurities and residual catalysts, thus guaranteeing the structure integrity. Second, ethanol’s wetting effect reduces axial friction between neighboring CNTs yet enhances the radial resistance, facilitating the alignment during direct assembly. Additionally, the rapid volatility of ethanol optimizes the voids among CNT bundles, further improving the uniformity of the CNT fibers.

Uniformity can be extensively affected by carrier gas flow management. Excessively high flow rates induce turbulence, thus leading to an uneven carrier gas distribution. Concomitantly, fluctuations occur for the CNT growth rates which result in inconsistent orientations and ultimately compromise the macroscopic properties of CNT fibers. Conversely, excessively low flow rates result in prolonged reactant residence times, thus raising the risk of carbon accumulation on the catalyst surface, which inhibits CNT elongation and exacerbates structural defects. Improper flow rates can also disrupt carbon source decomposition and the diffusion of intermediate products, further affecting the quality of the CNT growth. The implementation of precise control of carrier gas flow is critical for achieving uniform CNT production. Techniques such as optimizing the flow distribution and monitoring real-time atmospheric changes can stabilize the reaction environment, therefore increasing the consistency of CNT growth and the uniformity of the CNT fibers.

## 5 Applications and Advanced Directions

The future development of fiber materials is moving toward intelligence and multifunction.^72–74^ With high conductivity and high-strength, the advanced applications of CNT fibers are vast, such as flexible wearable devices, energy storage, biomedical engineering, and aerospace (Figure 4).

**Figure 4:**
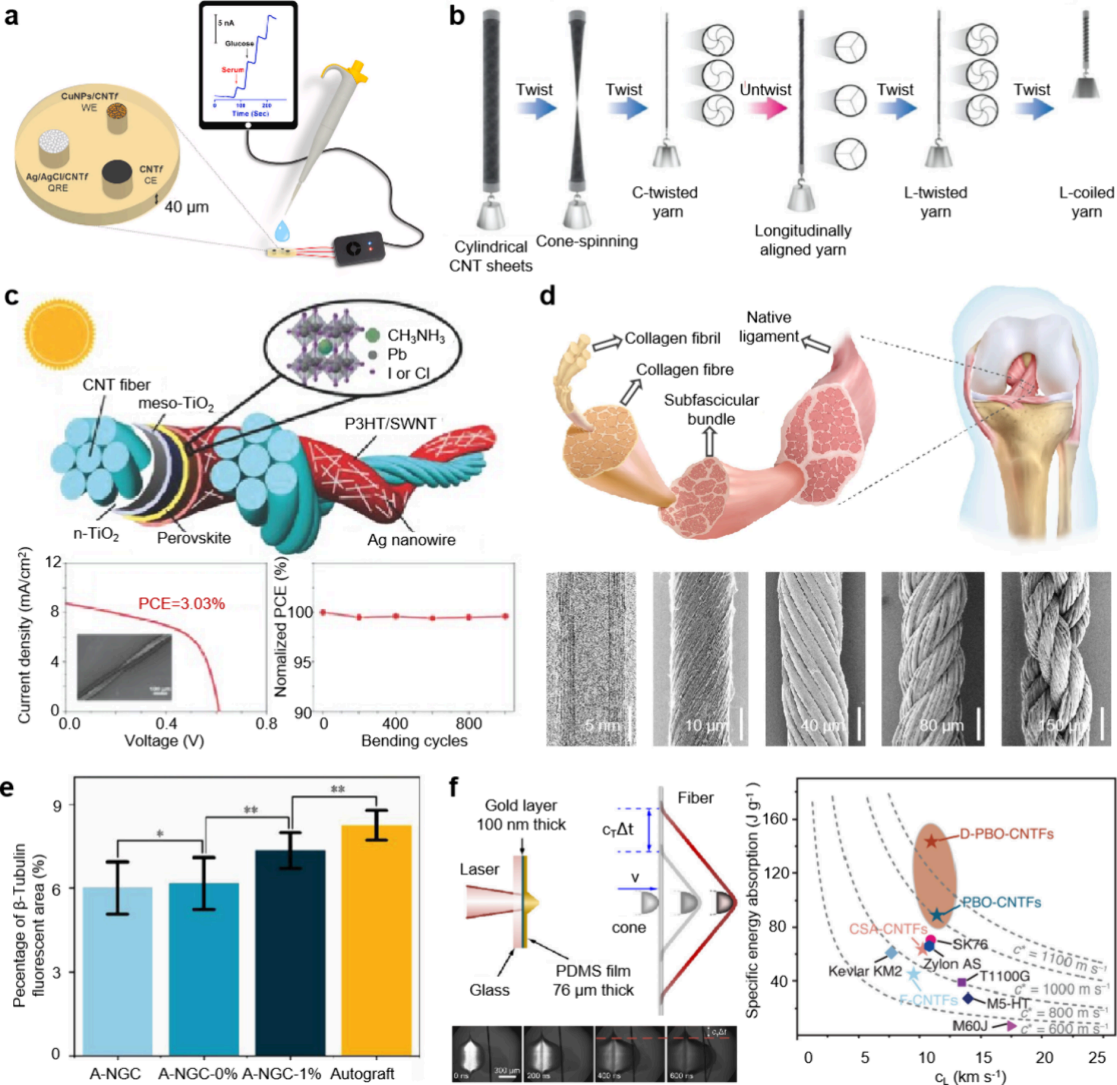
Applications of CNT fibers in various fields. (a) Highly sensitive nonenzymatic glucose sensor. Adapted with permission from ref.^75^ Copyright 2021 Elsevier. (b) Fabrication illustration of L-coiled fiber harvester. Adapted from ref.^76^ Available under a CC-BY (Creative Commons Attribution License) license. Copyright 2022 S. Oh et al. (c) Wearable double-twisted fiber perovskite solar cells with a maximum power conversion efficiency of 3.03% and superior bending stability. Adapted with permission from ref.^77^ Copyright 2015 John Wiley and Sons. (d) Bone-integrating anterior cruciate ligament replacement: structure of native ligament and hierarchical helical fibers made by CNTs. Adapted with permission from ref.^8^ Copyright 2023 Springer Nature. (e) Percentage of β-tubulin fluorescent area illustrates highly oriented artificial nerve guidance conduits loaded with MWCNTs exhibiting positive muscle nerve regeneration. Adapted with permission from ref.^78^ Copyright 2024 John Wiley and Sons. (f) High impact resistance: schematic diagram of the laser-induced high-velocity transverse impact on a fiber and comparison of specific energy absorption. Adapted with permission from ref.^16^ Copyright 2024 The American Association for the Advancement of Science.

### 5.1 Flexible Biomedical Sensors

CNT fibers exhibit high mechanical flexibility, high electrical conductivity, and good biocompatibility, making them ideal for flexible and wearable health-monitoring devices.^79^ These fibers maintain stable performances under bending, stretching, and twisting, enabling their integration into polymer matrices or textiles to develop high-sensitivity strain and pressure sensors. These sensors provide real-time monitoring of physiological parameters, including heart rate, respiration, and joint movements.^80^

In the realm of implantable biosensors, CNT fibers offer remarkable potential because of their large surface areas and high electrical conductivity, making them well-suited for detecting biochemical signals, such as glucose and lactate. For example, CNT fibers functionalized with copper oxide nanoparticles have demonstrated nonenzymatic glucose detection with exceptional sensitivity (up to 3000 μA/mM·cm^2^) and a detection limit as low as 1.4 μM (Figure 4a).^75,81^ Unlike traditional enzyme-based sensors, these devices exhibit superior stability and flexibility, minimizing implantation discomfort and mitigating the effects of environmental variables, such as pH, temperature, and humidity. Additionally, CNT fiber sensors resist common interferences, such as chloride ions and oxygen, ensuring reliable detection in complex biological environments.^82^ These advancements enable real-time monitoring of biofluids, including sweat, saliva, and interstitial fluid, providing efficient tools for the early diagnosis and management of chronic diseases such as diabetes.^83^

However, challenges remain in clinical translation. One major limitation is the long-term biocompatibility and stability of CNT fibers in vivo. While surface functionalization improves biointegration, concerns persist regarding potential inflammatory responses and degradation over extended implantation periods. Additionally, obtaining regulatory approval for CNT-based biomedical devices remains a significant hurdle as comprehensive pharmacological evaluations are required to determine long-term safety.

### 5.2 Energy Harvesting and Storage

CNT fibers are integral to energy harvesting devices. When integrated with thermoelectric materials, the composite demonstrated an enhanced energy conversion efficiency, transforming waste heat into usable electrical energy. Studies have reported that peak power outputs of 540 W·kg^–1^ and energy conversion efficiencies of 2.15% from torsional and tensile mechanical energy (Figure 4b).^76,84^ Furthermore, CNT fibers show promise in flexible solar cells, where they serve as transparent conductive electrodes or interconnects. The mechanical flexibility and high electrical conductivity of CNT fibers improve solar cell efficiency while preserving lightweight properties, which are critical for next-generation solar energy solutions.^85^ Recent studies also highlight the integration of CNT fibers with perovskites or other organic photovoltaics to establish high-efficiency and lightweight solar power generation (Figure 4c).^77,86^

Additionally, as electrodes in supercapacitors and lithium-ion batteries, CNT fibers demonstrate both high electrical conductivity and mechanical stability, and they are critical for improving device performance and durability.^87^ For example, CNT fibers decorated with MnO_2_ exhibit specific capacitances as high as 231.3 F/g in sodium-ion electrolytes, which is 23 times greater than unmodified CNT fibers. This modification significantly enhances the hydrophilicity and ionic conductivity of the electrodes, enabling the electrolyte to penetrate the fiber interior more effectively.

However, challenges remain in terms of scalability and long-term performance. The synthesis and processing of high-purity CNT fibers on an industrial scale are complex and costly, limiting widespread adoption. Furthermore, the stability of CNT-based electrodes in different electrolytes and under prolonged cycling conditions requires further optimization to mitigate degradation and ensure reliable long-term operation.

### 5.3 Biomedical Engineering

Owing to their unique combination of mechanical strength, flexibility, and electrical conductivity, CNT fibers are highly suitable for diverse biomedical applications. A particularly promising area is ligament and tendon repair, where CNT fibers mimic the anisotropic structure of natural ligaments, providing both strength and flexibility. Hierarchically helical CNT fibers (HHFs) have demonstrated superior performance in anterior cruciate ligament (ACL) reconstruction, enhancing graft-bone integration and reducing bone tunnel enlargement, outperforming conventional polymer grafts (Figure 4d).^8,88^ In rabbit models, these fibers enable complete bone tunnel repair, in contrast with the significant enlargement observed with clinical polymer grafts.

CNT fibers are also critical in neural repair. Their electrical conductivity facilitates the creation of nerve conduits that guide axon growth, while providing electrical stimulation to accelerate recovery. CNT-based scaffolds have shown promising results in peripheral nerve injury models, enhancing both structural regeneration and functional recovery (Figure 4e).^78,89^ Additionally, the high surface area of CNT fibers supports drug delivery systems, allowing therapeutic agents to attach to the fiber surface for targeted, sustained release.^90,91^ In tissue engineering, CNT-reinforced scaffolds combine mechanical strength with bioactivity, promoting cell growth and tissue integration in applications such as bone repair.^92,93^

However, significant barriers remain for clinical translation. The long-term biocompatibility of CNT fibers, particularly concerning potential immune responses, cytotoxicity, and degradation behavior, requires extensive in vivo validation. Further research is also needed to optimize functionalization strategies that enhance biointegration while ensuring long-term stability in physiological environments.

### 5.4 Aerospace and Space Exploration

CNT fibers hold significant potential for use in extreme environments, particularly in aerospace and space exploration. Advances in synthesis and post-treatment techniques have enabled CNT fibers to achieve tensile strengths exceeding 80 GPa, meeting stringent aerospace requirements.^94^ Studies have demonstrated their superior performance under high-strain-rate conditions which is critical for ballistic protection and debris shielding, where dynamic mechanical properties are essential (Figure 4f).^16^

Owing to their exceptional tensile strength and low density, CNT fibers are well suited for high-strength conductive cables. These lightweight, durable cables could potentially address challenges in reducing satellite and spacecraft payloads, while ensuring mechanical reliability. Furthermore, the excellent electrical conductivity of CNT fibers could also support efficient and reliable power transmission and data transfer in space environments.^95,96^

One visionary application of CNT fibers is their potential use in constructing a space elevator—a revolutionary concept in space transportation. CNT fibers uniquely combine the required tensile strength and low density to withstand immense stresses. With tensile strengths of up to 100 GPa and densities as low as 1.3 g/cm^3^, CNT fibers far surpass those of traditional materials.^16,94,97^

However, major challenges remain before such applications become feasible. The large-scale production of defect-free, ultrahigh-strength CNT fibers is still an ongoing challenge. Additionally, the effects of space radiation on the CNT fiber stability and mechanical performance require further exploration. Although still conceptual, ongoing advancements in CNT fiber synthesis, post-treatment, and scalable production are steadily bringing this bold vision closer to reality.^98,99^

## 6 Conclusion

Owing to their unparalleled combination of tensile strength, lightweight nature, and superior electrical conductivity, CNT fibers are expected to transform industries in the fields of flexible electronics, energy storage, biomedicine, and aerospace. However, despite these merits, the widespread adoption of CNT fibers remains constrained by challenges related to micro- and macroscale performance optimization and continuous production scalability. Factors such as weak intertube interactions, misalignment, and structural defects hinder the efficient transfer of extraordinary intrinsic properties of individual CNTs to macroscopic CNT fibers, indicating a notable performance disparity between the microscale and macroscale. While the FCCVD method offers promising avenues for large-scale production, achieving consistent quality, uniformity, and scalability continues to pose a few technical hurdles. Future research should focus on refining reactor design, optimizing catalyst composition, and advancing in-line quality control techniques to ensure scalable fiber production with minimal defects. Additionally, interdisciplinary efforts in machine learning, automation, and sustainable material sourcing will be crucial to enhancing the production efficiency and commercial viability. Addressing these challenges is essential to unlocking the full potential of CNT fibers for next-generation applications.

Advancements in fabrication techniques are pivotal to overcoming these barriers. Refining catalyst designs, enhancing fiber alignment during FCCVD production, and implementing advanced postprocessing methods are crucial for improving the structural integrity and performance of the CNT fibers. Additionally, integrating machine learning with real-time monitoring and control technologies provides transformative capabilities for optimizing production processes, ensuring scalability without compromising the unique properties of CNT fibers. With sustained research and development, CNT fibers are well-positioned to drive innovation across diverse fields. By addressing current limitations and leveraging emerging technologies, CNT fibers have the potential to enable groundbreaking applications, paving the way for transformative advancements in next-generation materials and technologies.

## Supplementary Material



## Notes

Transparent Peer Review report available (PDF)
