# Supplementary material for: Advancing Carbon Nanotube Fibers: Addressing Challenges from Production to Application
Source: ACS Cent Sci. 2025 Jun 3;11(6):855–67. doi: 10.1021/acscentsci.5c00155 (PMC12203435; doi:10.1021/acscentsci.5c00155)
Supplement: Supplementary file 1 [file oc5c00155_si_001.pdf]

oc-2025-00155p.R1

Name: Peer Review Information for "Advancing Carbon Nanotube Fibers: Addressing Challenges from Production to Application"

First Round of Reviewer Comments

Reviewer: 1

Comments to the Author

Reviewer Report

1. General Comments

The manuscript titled "Advancing Carbon Nanotube Fibers: Addressing Challenges from Production to Application" provides a comprehensive overview of the current state of carbon nanotube (CNT) fiber research, focusing on the challenges and opportunities in their production and application. The authors have done a proper job of summarizing the key issues related to CNT fiber fabrication, including microscale and macroscale challenges, and have highlighted potential solutions and future directions. The paper is well-structured and provides valuable insights into the field. However, there are areas where the manuscript could be improved to enhance clarity, depth, and overall impact.

2. Specific Comments by Section

2.1 Abstract

- Strengths: The abstract provides a concise summary of the paper's focus, including the challenges and potential applications of CNT fibers.
- Suggestions:
  - Consider adding a brief mention of the key findings or breakthroughs discussed in the paper to give readers a clearer sense of the paper's contributions.
  - The abstract could benefit from a more explicit statement of the paper's novelty or unique perspective compared to existing reviews in the field.

2.2 Introduction

- Strengths: The introduction effectively sets the stage for the discussion by outlining the properties and potential applications of CNT fibers. It also highlights the challenges in translating the properties of individual CNTs to macroscopic fibers.
- Suggestions:
  - The introduction could be strengthened by providing a more detailed comparison of CNT fibers with other advanced materials (e.g., graphene fibers, carbon fibers) to contextualize their unique advantages and limitations.
  - A brief discussion of the historical development of CNT fiber research would help readers understand the evolution of the field.

2.3 Microscale Challenges

- Strengths: This section provides a thorough discussion of the challenges at the microscale, including reactor design, catalyst optimization, and growth process regulation. The inclusion of computational fluid dynamics (CFD) simulations and experimental results adds depth to the discussion.
- Suggestions:
  - The section could benefit from a more detailed discussion of the limitations of current reactor designs and catalyst systems. For example, what are the specific bottlenecks in achieving uniform CNT growth at scale?
  - The authors should consider adding a table summarizing the key parameters (e.g., temperature, gas flow rate, catalyst type) and their effects on CNT growth, as this would help readers quickly grasp the critical factors.

2.4 Macroscale Challenges

- Strengths: The discussion of mechanical processing, heat treatment, and modification treatments is comprehensive and well-supported by recent research findings. The inclusion of schematics and experimental data enhances the clarity of the discussion.
- Suggestions:
  - The section could be improved by providing a more detailed comparison of the different mechanical processing techniques (e.g., twisting, stretching, rolling) in terms of their effectiveness in improving fiber properties.

o The authors should consider adding a discussion of the trade-offs between different post-treatment methods (e.g., heat treatment vs. polymer infiltration) in terms of cost, scalability, and performance.

2.5 Challenges in CNT Production

• Strengths: This section effectively addresses the challenges of continuous production and uniformity, which are critical for the industrial application of CNT fibers. The discussion of machine learning (ML) integration is particularly insightful and highlights a promising direction for future research.

• Suggestions:

o The authors should provide more details on the specific ML models and algorithms used in CNT fiber production, as well as their limitations and potential for further improvement.

o A discussion of the economic feasibility of scaling up CNT fiber production using current methods would be valuable, as this is a key consideration for industrial adoption.

2.6 Applications and Advanced Directions

• Strengths: The section provides a broad overview of the potential applications of CNT fibers, including flexible biomedical sensors, energy harvesting, biomedical engineering, and aerospace. The inclusion of specific examples and case studies adds depth to the discussion.

• Suggestions:

o The authors should consider adding a more critical analysis of the current limitations of CNT fibers in each application area. For example, what are the key barriers to their adoption in flexible electronics or aerospace?

o A discussion of the regulatory and safety considerations for CNT fibers in biomedical applications would be valuable, as this is a critical issue for their commercialization.

2.7 Conclusion

• Strengths: The conclusion effectively summarizes the key challenges and opportunities in CNT fiber research and highlights the importance of continued innovation in this field.

• Suggestions:

o The conclusion could be strengthened by providing a more explicit roadmap for future research, including specific areas where breakthroughs are needed (e.g., catalyst design, post-treatment methods).

o The authors should consider adding a brief discussion of the potential societal and economic impacts of widespread CNT fiber adoption, as this would help contextualize the importance of the research.

3. Overall Assessment

• Strengths: The manuscript provides a comprehensive and well-organized overview of the challenges and opportunities in CNT fiber research. The inclusion of recent experimental results and computational studies adds depth to the discussion, and the paper is well-supported by references to the latest literature.

• Weaknesses: While the paper is strong overall, there are areas where it could be improved, particularly in terms of providing more detailed comparisons, critical analysis, and future directions. The discussion of economic and regulatory considerations is also somewhat limited.

4. Recommendation

Assessment: Major Revision

The paper is well-written and provides valuable insights into the field of CNT fiber research. However, it would benefit from some additional details and critical analysis in certain sections, particularly in the discussion of applications and future directions. With these revisions, the paper will be suitable for publication.

5. Suggested Revisions

1. Abstract: Add a brief mention of key findings or breakthroughs to highlight the paper's contributions.

2. Introduction: Include a comparison with other advanced materials and a brief historical overview of CNT fiber research.

3. Microscale Challenges: Add a table summarizing key parameters and their effects on CNT growth.

4. Macroscale Challenges: Provide a more detailed comparison of mechanical processing techniques and discuss trade-offs between post-treatment methods.

5. Challenges in CNT Production: Include more details on ML models and algorithms, and discuss the economic feasibility of scaling up production.

6. Applications and Advanced Directions: Add a critical analysis of current limitations in each application area and discuss regulatory and safety considerations.

7. Conclusion: Provide a more explicit roadmap for future research and discuss the potential societal and economic impacts of CNT fiber adoption.

With these revisions, the manuscript will be significantly strengthened and ready for publication.

Reviewer: 2

Comments to the Author

This manuscript provides a systematic review of the production and application of carbon nanotube fibers (CNT fibers) in the field of materials science, offering a comprehensive discussion on the current challenges and potential solutions for achieving continuous and uniform production of CNT fibers. The paper is well-structured, logically presented, and well written.

Therefore, I suggest this article could be accepted after the author addresses the following questions.

1. While this review focused on the production and application of carbon nanotube (CNT) fibers, the fabrication techniques discussed are primarily centered on the floating catalyst chemical vapor deposition (FCCVD). Other methods were briefly mentioned and the distinct advantages of FCCVD over these alternative techniques were not sufficiently highlighted. It is recommended to include a comparative table to evaluate various fabrication techniques based on key parameters such as continuity, uniformity, production scalability, cost-efficiency, tensile strength, and electrical conductivity.

2. Biomass-derived carbon source was mentioned in this review. It is anticipated to make a more detailed evaluation of their performance and potential industrial application. Specifically, a deeper exploration of their feasibility as a sustainable alternative to petroleum-based carbon source would significantly enhance the discussion.

3. This manuscript introduced optimizations of FCCVD reactor designs, carbon sources, catalyst precursor materials and growth process. It would be beneficial to extend

the discussion on the application of these optimization within industrial contexts. Additionally, addressing the gap between theoretical advancements and practical implementation would provide valuable insights for readers.

4. Machine learning (ML) is briefly mentioned as a tool for optimizing CNT fibers production. A deeper discussion is anticipated to explain the process of adjusting reaction conditions by optimizing model parameters dynamically. Furthermore, a comparison of ML-based approaches with traditional methods, particularly in terms of efficiency improvements, would strengthen the manuscript.

5. This manuscript addresses challenges and potential solutions for CNT fibers at macroscale levels. A more detailed analysis focusing on the synergistic and antagonistic effects of these proposed methods is expected to offer valuable insights.

6. It would be beneficial to consult more recent work about high performance fibers and their applications. The works on high performance fibers (SusMat. 2024, 4, e205; eScience, 2024, 4, 100250; Adv Mater., 2024, 2407009; Adv. Fiber Mater. 2022, 4, 1572–1583.) could be used as reference.

Reviewer: 3

Comments to the Author

This review has shown the recent progress and potential challenges in CNT fiber research from production to application, providing valuable guidance and insights for the CNT fiber research community. I would like to recommend the acceptance of paper after a minor revision.

1) Regarding the floating CVD based continuous growth of CNTs, although the authors have presented some progress in dynamic reaction simulations, catalyst design, carbon source optimization, and growth mechanism etc., it would be beneficial to add some recent breakthroughs in catalyst designs and growth rate control in continuous synthesis processes.

2) Concerning the CNT fiber property enhancement, the authors addressed the importance of the cross-scale assembly challenges, it's better to make a comparative analysis of different interface engineering routes (e.g., covalent functionalization vs. π-π interactions)

and their respective strengthening efficiencies, providing instructive interfacial regulating strategies toward creative applications.

Author's Response to Peer Review Comments:

Dear Reviewers,

We sincerely appreciate the time and effort you have dedicated to reviewing our manuscript (oc-2025-00155p). We are especially grateful for the constructive comments from the three reviewers, which have guided us in making further improvements and revisions, strengthening our manuscript through more in-depth and detailed discussions. These revisions have allowed us to fully justify the significance of our work. We are highly grateful for the appreciation of our work from

Reviewer #1 as “The manuscript titled 'Advancing Carbon Nanotube Fibers: Addressing Challenges from Production to Application' provides a comprehensive overview of the current state of carbon nanotube (CNT) fiber research, focusing on the challenges and opportunities in their production and application. The authors have done a proper job of summarizing the key issues related to CNT fiber fabrication, including microscale and macroscale challenges, and have highlighted potential solutions and future directions. The paper is well-structured and provides valuable insights into the field”.

Reviewer #2 as “This manuscript provides a systematic review of the production and application of carbon nanotube fibers (CNT fibers) in the field of materials science, offering a comprehensive discussion on the current challenges and potential solutions for achieving continuous and uniform production of CNT fibers. The paper is well-structured, logically presented, and well written. Therefore, I suggest this article could be accepted after the author addresses the following questions”.

Reviewer #3 as “This review has shown the recent progress and potential challenges in CNT fiber research from production to application, providing valuable guidance and insights for the CNT fiber research community. I would like to recommend the acceptance of paper after a minor revision”.

Following the reviewers' insightful suggestions, we have conducted additional detailed discussions, and carefully revised the manuscript to improve clarity and precision. All changes are highlighted in blue in the revised versions of the Manuscript. Given our comprehensive responses and the thorough revisions made, we believe that all concerns raised by the reviewers have been fully addressed. We remain open to any further inquiries

and sincerely appreciate your time and effort in re-evaluating our work. For further clarity, we have also prepared a point-by-point response document addressing each comment in detail.

Thank you very much again!

Yours sincerely,

Huisheng PENG, The University Professor

Department of Macromolecular Science, Laboratory of Advanced Materials

Fudan University, Shanghai 200438, China

Phone: 86-21-31242850; Email: penghs@fudan.edu.cn

Webpage: www.fiber.fudan.edu.cn

Response to Reviewers

(*black italic: Reviewer's comment*; black type: Our response; blue type: Key revisions)

Technical Responses to Reviewer #1

Comment #0. *The manuscript titled 'Advancing Carbon Nanotube Fibers: Addressing Challenges from Production to Application' provides a comprehensive overview of the current state of carbon nanotube (CNT) fiber research, focusing on the challenges and opportunities in their production and application. The authors have done a proper job of summarizing the key issues related to CNT fiber fabrication, including microscale and macroscale challenges, and have highlighted potential solutions and future directions. The paper is well-structured and provides valuable insights into the field. The abstract provides a concise summary of the paper's focus, including the challenges and potential applications of CNT fibers. Consider adding a brief mention of the key findings or breakthroughs discussed in the paper to give readers a clearer sense of the paper's contributions. The abstract could benefit from a more explicit statement of the paper's novelty or unique perspective compared to existing reviews in the field. The introduction effectively sets the stage for the discussion by outlining the properties and potential applications of CNT fibers. It also highlights the challenges in translating the properties of individual CNTs to macroscopic fibers. The introduction could be strengthened by providing a more detailed comparison of CNT fibers with other advanced materials (e.g., graphene fibers, carbon fibers) to contextualize their unique advantages and limitations. A brief discussion of the historical development of CNT fiber research would help readers understand the evolution of the field. This section provides a thorough discussion of the challenges at the microscale, including reactor design, catalyst optimization, and growth process regulation. The inclusion of computational fluid dynamics (CFD) simulations and experimental results adds depth to the discussion. The section could benefit from a more detailed discussion of the limitations of current reactor designs and catalyst systems. For example, what are the specific bottlenecks in achieving uniform CNT growth at scale? The authors should consider adding a table summarizing the key parameters (e.g., temperature, gas flow rate, catalyst type) and their effects on CNT growth, as this would help readers quickly grasp the critical factors. The discussion of mechanical processing, heat treatment, and modification treatments is comprehensive and well-supported by recent research findings. The inclusion of schematics and experimental data enhances the clarity of the discussion. The section could be improved by providing a more detailed comparison of the different mechanical processing techniques (e.g., twisting, stretching, rolling) in terms of their effectiveness in improving fiber properties. The authors should consider adding a discussion of the trade-offs between different post-treatment methods (e.g., heat treatment vs. polymer infiltration) in terms of cost, scalability, and performance. This section effectively addresses the challenges of continuous production and uniformity, which are critical for the industrial application of CNT fibers. The discussion of machine learning (ML) integration is particularly insightful and highlights a promising direction for future research. The authors should provide more details on the specific ML models and algorithms used in CNT fiber production, as well as their limitations and potential for further improvement. A discussion of*

the economic feasibility of scaling up CNT fiber production using current methods would be valuable, as this is a key consideration for industrial adoption. The section provides a broad overview of the potential applications of CNT fibers, including flexible biomedical sensors, energy harvesting, biomedical engineering, and aerospace. The inclusion of specific examples and case studies adds depth to the discussion. The authors should consider adding a more critical analysis of the current limitations of CNT fibers in each application area. For example, what are the key barriers to their adoption in flexible electronics or aerospace? A discussion of the regulatory and safety considerations for CNT fibers in biomedical applications would be valuable, as this is a critical issue for their commercialization. The conclusion effectively summarizes the key challenges and opportunities in CNT fiber research and highlights the importance of continued innovation in this field. The conclusion could be strengthened by providing a more explicit roadmap for future research, including specific areas where breakthroughs are needed (e.g., catalyst design, post-treatment methods). The authors should consider adding a brief discussion of the potential societal and economic impacts of widespread CNT fiber adoption, as this would help contextualize the importance of the research. The manuscript provides a comprehensive and well-organized overview of the challenges and opportunities in CNT fiber research. The inclusion of recent experimental results and computational studies adds depth to the discussion, and the paper is well-supported by references to the latest literature. While the paper is strong overall, there are areas where it could be improved, particularly in terms of providing more detailed comparisons, critical analysis, and future directions. The discussion of economic and regulatory considerations is also somewhat limited. The paper is well-written and provides valuable insights into the field of CNT fiber research. However, it would benefit from some additional details and critical analysis in certain sections, particularly in the discussion of applications and future directions. With these revisions, the paper will be suitable for publication.

Response #0: We sincerely thank you for evaluating our manuscript and the recommendation for publication after major revision. In the following section, we addressed each comment with a detailed explanation and supplement. We believe these changes consolidate the technical discussions. For your convenience, the main revisions are marked in blue font in the revised manuscript.

Comment #1. *Abstract: Add a brief mention of key findings or breakthroughs to highlight the paper's contributions.*

Response #1: We sincerely thank the reviewer for the professional question. Our outlook provides a systematic examination of the challenges and advancements in CNT fiber production, analyzing key factors that influence performance from the nanoscale to the macroscale. We discuss recent innovations in synthesis techniques, material optimization, and

structural control, offering a comprehensive overview of the field's current progress. In particular, we highlight the growing role of machine learning in optimizing production parameters and the significant impact of airflow control on fiber uniformity and scalable manufacturing. To improve clarity, we have revised the abstract to concisely summarize the key breakthroughs presented in this outlook. Specifically, we have emphasized the novelty of our approach, which integrates machine learning with process optimization strategies to enhance CNT fiber production. By combining advanced computational methods with manufacturing efficiency, our work offers a unique perspective that differentiates it from existing reviews.

Action #1: The revised abstract is presented below:

"This outlook highlights the importance of innovative strategies for multiscale performance optimization and the advances in scalable fabrication methodologies. A distinctive perspective on CNT fiber production is provided, emphasizing the integration of machine learning with process optimization strategies to enhance uniformity and efficiency."

Comment #2. *Introduction: Include a comparison with other advanced materials and a brief historical overview of CNT fiber research.*

Response #2: We sincerely appreciate the reviewer's valuable suggestions. To enhance the introduction, we have incorporated a comparative analysis of CNT fibers with other advanced materials, specifically carbon and graphene fibers. This comparison highlights the distinctive properties of CNT fibers and underscores their potential for multifunctional applications. Additionally, we have emphasized the critical need for scalable fabrication methods to fully leverage these advantages.

Action #2: The revised introduction is presented below:

(*Paragraph 2* on *Page 4* in the revised manuscript)

"Compared to conventional carbon and graphene fibers, CNT fibers uniquely integrate high electrical conductivity, mechanical robustness, and flexibility.[11, 12] While carbon fibers are widely utilized in aerospace and automotive industries, their electrical conductivity remains relatively low.[13] Graphene fibers, on the other hand, exhibit exceptional conductivity but face challenges in mechanical performance and large-scale production.[14, 15] CNT fibers offer a well-balanced combination of strength, conductivity, and lightweight properties, positioning them as promising candidates for multifunctional applications, such as structural electronics

and smart textiles.[16] However, the practical realization of these advantages hinges on the development of scalable fabrication techniques that enable precise control over CNT alignment, purity, and structural integrity. Advancing these production methods is therefore crucial to fully unlocking the potential of CNT fibers."

Comment #3. *Microscale Challenges: Add a table summarizing key parameters and their effects on CNT growth.*

Response #3: We sincerely appreciate the reviewer's insightful suggestion. While industrial-scale production remains in its early stages, our work does not overlook this critical aspect. Rather, we emphasize the necessity of first establishing small-scale experimental models in the laboratory. These controlled studies enable a systematic investigation of key parameters, providing essential insights that can later guide the scaling-up process. To strengthen this discussion, we have further refined our analysis to better illustrate the transition from fundamental research to practical application. Additionally, we have incorporated Table 2, which systematically summarizes the key parameters influencing CNT growth, their effects on CNT fiber properties, and corresponding optimization strategies. This table provides a clear and structured overview of how various factors impact fiber quality and production outcomes.

Action #3: The Table 2 is presented below:

(*On Page 12* in the revised manuscript)

Table 2. Properties of CNT Fibers at Different Growth Conditions

**Growth Parameter**	**Key Characteristics and Significance**	**Effects on CNT Fiber Properties**	**Optimization Strategies**
Growth Temperature (°C)	1000–1300 °C (Typical FCCVD range)	Low yield, structural defects (low T); catalyst deactivation, amorphous carbon (high T)[26, 44]	High-temperature-stable catalysts (MOF-derived); thermal field optimization
Gas Flow Rate (sccm)	500–5000 sccm (Depending on reactor size and CNT production needs)	Catalyst aggregation, misalignment (low flow); turbulence, fiber irregularity (high flow)[29, 45]	CFD simulations; reactor design optimization
Catalyst Type	Fe, Co, Ni, MOF-derived catalysts	CNT diameter, crystallinity, length variation[46, 47]	MOF-derived catalysts; defect reduction

Carbon Source	Ethanol, toluene, methane, biomass	CNT yield, tube diameter, defect density variation[23, 48]	Multi-component carbon sources; yield enhancement
Reactor Configuration	Horizontal and vertical reactors	Horizontal: More High turbulence, misalignment (horizontal); improved uniformity (vertical)[23, 49]	Vertical reactor design; turbulence minimization

Comment #4. *Macroscale Challenges: Provide a more detailed comparison of mechanical processing techniques and discuss trade-offs between post-treatment methods.*

Response #4: We thank the reviewer for this valuable question. To address the trade-offs between post-treatment methods, we have conducted a comprehensive analysis of how various techniques influence CNT alignment, packing density, and structural integrity. Additionally, we have expanded our discussion on mechanical processing techniques by detailing four widely used methods:

Drawing: Enhances CNT alignment and improves tensile properties but may introduce structural defects.

Twisting: Improves fiber uniformity and strength utilization but can lead to performance reduction due to excessive torsion.

Stretching: Increases CNT alignment and tensile strength but requires precise control to avoid breakage.

Rolling: Enhances packing density, mechanical strength, and conductivity, though agglomeration remains a challenge.

Each method plays a crucial role in optimizing CNT fiber performance, yet they also present specific limitations that must be carefully managed. Furthermore, we provide a brief comparison of additional post-treatment strategies, including chemical doping, polymer infiltration, and thermal annealing. These methods further modify CNT fiber properties by enhancing electrical conductivity, mechanical strength, and long-term stability.

Action #4: The Table 3 is presented below:

(*On Page 16* in the revised manuscript)

Table 3. Comparison of Mechanical Processing Techniques for CNT fibers

**Method**	**Effect on CNT fibers**	**Challenges**
Drawing	CNT alignment, tensile enhancement[50, 56]	Lowering structural defects

Twisting	Uniformity, strength utilization[59, 60]	Avoiding performance reduction
Stretching	CNT alignment, tensile strength[62, 63]	Precise stretching control
Rolling	Increased packing density, strength, conductivity[57, 64]	Agglomeration, flexibility reduction

Comment #5. *Challenges in CNT Production: Include more details on ML models and algorithms, and discuss the economic feasibility of scaling up production.*

Response #5: Thank you for your insightful comments. Machine learning (ML) requires a substantial amount of high-quality data for effective model training, while the floating catalyst chemical vapor deposition (FCCVD) process is complex, time-consuming, and challenging to operate. Consequently, only two published studies have explored the application of ML in CNT fiber production using the FCCVD method. Most ML models and algorithms aimed at optimizing CNT growth are based on the array growth process, which shares some similarities with FCCVD. Despite these challenges, ML has demonstrated its ability to significantly enhance experimental efficiency and accelerate research timelines. To provide a more detailed discussion, we have expanded our analysis of ML models and their potential applications in CNT fiber production. Additionally, we have addressed the economic feasibility of scaling up production by discussing how ML-driven optimization can reduce costs and improve process efficiency.

Action #5: The following text has been improved to the revised manuscript:

(*Paragraph 3, 4* on *Page 11* in the revised manuscript Chapter 2.3)

"Due to the numerous variables involved in the CNT production process, traditional trial-and-error experimental methods are insufficient for optimizing parameters. Recent studies have introduced machine learning (ML) techniques to navigate the multidimensional parameter space, identify optimal synthesis conditions, and facilitate the development of CNTs with specific properties. Multiparameter optimization models establish correlations between synthesis parameters (e.g., carbon feed, temperature, and catalyst amount) and CNT properties, enabling predictive control over fiber quality, yield, and scalability. However, the application of ML in FCCVD-based CNT synthesis remains limited. Most ML models are designed for the CVD method used in CNT array growth, which shares some similarities with FCCVD. ML algorithms—including artificial neural networks (ANN), random forests (RF), support vector machines (SVM), eXtreme gradient boosting (XGBoost), and Bayesian optimization (BO)—have demonstrated high accuracy in modeling CNT array growth.[38-41]

Furthermore, ML has been shown to significantly improve experimental efficiency and shorten research timelines. For instance, dynamic optimization via BO accelerated experimental speed by more than five times.[42] When combined with automation, over 500 experiments and millions of virtual simulations were completed within just 43 days, achieving the highest precision in producing HACNT arrays at a specified density.[43] From an economic perspective, integrating ML into industrial-scale CNT production offers promising advantages. ML-driven optimization can help unlock the complex growth mechanisms in FCCVD, bridge the performance gap between experimental and theoretical values, and reduce production costs and time compared to traditional methods. These advancements are expected to accelerate the industrialization of CNT fibers, making scalable manufacturing more economically viable."

Comment #6. *Applications and Advanced Directions: Add a critical analysis of current limitations in each application area and discuss regulatory and safety consideration.*

Response #6: Thank you very much for the constructive and professional suggestion. To enhance our discussion, we have revised the manuscript to provide a critical analysis of the key limitations in each application area, as well as an overview of regulatory and safety considerations. The primary challenges include: **Biomedical applications:** Long-term biocompatibility concerns, particularly regarding CNT toxicity, immune response, and degradation over extended implantation periods. Regulatory approval remains a significant hurdle due to stringent pharmacological evaluations required for long-term safety. **Energy applications:** Scalability and stability challenges, as the complex and costly synthesis of high-purity CNT fibers limits large-scale adoption. Additionally, CNT-based electrodes require further optimization to maintain stability in different electrolytes and under prolonged cycling conditions. **Aerospace applications:** Structural durability and resistance to space radiation, as exposure to extreme conditions may degrade CNT fibers over time. Rigorous mechanical and radiation shielding tests are essential before deployment in aerospace environments.

Additionally, we have expanded our discussion on safety and regulatory considerations: **Biomedical applications** must meet strict approval processes due to potential cytotoxicity and long-term interactions with biological systems. **Energy applications** require extensive performance validation to ensure reliability, environmental compliance, and sustainability. **Aerospace applications** must undergo rigorous mechanical and radiation resistance testing to

confirm durability in extreme operational conditions.

Action #6: The following text has been improved to the revised manuscript:

In section 5.1: "However, challenges remain in clinical translation. One major limitation is the long-term biocompatibility and stability of CNT fibers in vivo. While surface functionalization improves biointegration, concerns persist regarding potential inflammatory responses and degradation over extended implantation periods. Additionally, regulatory approval for CNT-based biomedical devices remains a hurdle, as comprehensive pharmacological evaluation to determine its long-term safety."

In section 5.2: "However, challenges remain in scalability and long-term performance. The synthesis and processing of high-purity CNT fibers at an industrial scale are complex and costly, limiting widespread adoption. Furthermore, the stability of CNT-based electrodes in different electrolytes and under prolonged cycling conditions requires further optimization to mitigate degradation and ensure reliable long-term operation."

In section 5.3: "However, significant barriers remain for clinical translation. The long-term biocompatibility of CNT fibers, particularly concerning potential immune responses, cytotoxicity, and degradation behavior, requires extensive in vivo validation. Further research is also needed to optimize functionalization strategies that enhance biointegration while ensuring long-term stability in physiological environments."

In section 5.4: "However, major challenges remain before such applications become feasible. The large-scale production of defect-free, ultra-high-strength CNT fibers is still an ongoing challenge. Additionally, the effects of space radiation on CNT fiber stability and mechanical performance require further exploration. Although still conceptual, ongoing advancements in CNT fiber synthesis, post-treatment, and scalable production are steadily bringing this bold vision closer to reality."

***Comment** #7. Conclusion: Provide a more explicit roadmap for future research and discuss the potential societal and economic impacts of CNT fiber adoption.*

Response #7: Thank you for your insightful comments. To strengthen the conclusion, we have revised this section to provide a clearer roadmap for future research while also addressing the societal and economic impacts of CNT fiber adoption. Given the rapidly evolving nature of CNT fiber research, we have identified critical areas for advancement that will guide future developments without the need for an overly detailed roadmap.

Action #7: The revised instruction is presented below:

"Future research should focus on refining reactor design, optimizing catalyst composition, and advancing in-line quality control techniques to ensure scalable fiber production with minimal defects. Additionally, interdisciplinary efforts in machine learning, automation, and sustainable material sourcing will be crucial to enhancing production efficiency and commercial viability. Addressing these challenges is essential to unlocking the full potential of CNT fibers for next-generation applications."

Technical Responses to Reviewer #2

Comment #0. *This manuscript provides a systematic review of the production and application of carbon nanotube fibers (CNT fibers) in the field of materials science, offering a comprehensive discussion on the current challenges and potential solutions for achieving continuous and uniform production of CNT fibers. The paper is well-structured, logically presented, and well written. Therefore, I suggest this article could be accepted after the author addresses the following questions.*

Response #0: Thank you for your positive feedback and the recommendation to accept the manuscript. We appreciate your recognition of the paper's structure, presentation, and writing quality. We have carefully addressed the questions and suggestions you raised to improve the manuscript further. For your convenience, the main revisions are highlighted in blue font in the revised manuscript.

Comment #1. *While this review focused on the production and application of carbon nanotube (CNT) fibers, the fabrication techniques discussed are primarily centered on the floating catalyst chemical vapor deposition (FCCVD). Other methods were briefly mentioned and the distinct advantages of FCCVD over these alternative techniques were not sufficiently highlighted. It is recommended to include a comparative table to evaluate various fabrication techniques based on key parameters such as continuity, uniformity, production scalability, cost-efficiency, tensile strength, and electrical conductivity.*

Response #1: Thank you for evaluating our manuscript and we greatly appreciate your valuable suggestions. Carbon nanotube (CNT) fibers can be fabricated using various methods, including wet spinning, array spinning, and floating catalyst chemical vapor deposition (FCCVD). In practical applications, achieving both large-scale production and superior material performance is of primary importance. To address this, we have included a comparative table that evaluates these three fabrication techniques based on key parameters, such as uniformity, tensile strength, electrical conductivity, production scalability, cost-efficiency, and environmental impact. The revised discussion highlights the distinct advantages of FCCVD over other techniques. Specifically, FCCVD demonstrates lower production costs, higher performance, and a more environmentally friendly process, making it a promising candidate for industrial-scale CNT fiber manufacturing.

Action #1: The Table 1 is presented below:

(*Table 1* on *Page 5* in the revised manuscript)

Table 1. The comparison of various fabrication methods

Method	Uniformity	Tensile strength	Electrical conductivity	Production scalability	High cost-efficiency	Environmentally friendly
Wet spinning	★	★	★	★★	★	★
Array spinning	★★★	★★★	★★★	★	★	★★
FCCVD	★★	★★★	★★★	★★★	★★★	★★★

Note: A higher number of stars indicates better performance in the respective category. The maximum rating is ★★★, representing the best performance.

***Comment* #2.** *Biomass-derived carbon source was mentioned in this review. It is anticipated to make a more detailed evaluation of their performance and potential industrial application. Specifically, a deeper exploration of their feasibility as a sustainable alternative to petroleum-based carbon source would significantly enhance the discussion.*

Response #2: Thank you for your insightful comments. While previous studies have primarily focused on liquid and gaseous carbon sources for CNT fiber production, biomass-derived carbon sources have received less attention due to the need for additional processing, as they are solid in nature. However, biomass carbon sources offer significant advantages, including their renewability and low cost, making them an attractive alternative to petroleum-based sources. To further enhance our discussion, we have added a more detailed evaluation of biomass carbon sources, specifically exploring their feasibility as a sustainable alternative to traditional petroleum-based carbon sources. Biomass carbon sources can be derived from agricultural and forestry waste, which not only lowers production costs but also contributes to waste reduction. Additionally, the surfaces of these carbon sources are rich in functional groups and heteroatoms, which may improve the performance of CNT fibers.

Action #2: The revised manuscript is presented below:

(*Paragraph 2* on *Page 10* in the revised manuscript Chapter 2.2)

"Biomass-derived carbon sources hold significant potential for industrial applications. As a renewable resource, they can be obtained from agricultural and forestry waste, thereby reducing production costs.[35] Additionally, their surfaces are rich in functional groups and heteroatoms, which may enhance the performance of CNT fibers.[36] Therefore, biomass carbon sources are expected to play a vital role in the future industrial production of CNT fibers."

Comment #3. *This manuscript introduced optimizations of FCCVD reactor designs, carbon sources, catalyst precursor materials and growth process. It would be beneficial to extend the discussion on the application of these optimization within industrial contexts. Additionally, addressing the gap between theoretical advancements and practical implementation would provide valuable insights for readers.*

Response #3: Huge thanks to the reviewer for the valuable suggestions. We have expanded the manuscript to include a more detailed discussion of the challenges and prospects associated with applying the optimizations mentioned—FCCVD reactor designs, carbon sources, catalyst precursor materials, and growth processes—within an industrial context. We address several key challenges in scaling up FCCVD optimization for industrial use:

Reactor Design and Temperature Control: While reducing turbulence and controlling temperature gradients have proven effective in small-scale experiments, maintaining these parameters in large-scale reactors remains challenging. The complex gas dynamics in industrial reactors often lead to inconsistent CNT growth, which negatively impacts fiber uniformity and mechanical performance. To address this, we suggest incorporating computational fluid dynamics (CFD) simulations and real-time process monitoring to stabilize production conditions. These strategies will help optimize flow dynamics, reduce turbulence-induced inconsistencies, and improve the uniformity of CNT fibers at an industrial scale.

Carbon Source and Catalyst Optimization: While multi-component carbon sources have shown promise in enhancing CNT growth, their industrial application faces challenges related to cost, stability, and byproduct generation. Additionally, MOF-derived catalysts demonstrate high efficiency but require further development to address large-scale synthesis and long-term stability. We recommend focusing on the development of cost-effective, high-stability catalysts and optimizing continuous catalyst feeding strategies to ensure consistent CNT growth in industrial production.

Addressing the Gap Between Lab and Industrial Production: Laboratory studies often optimize individual variables under controlled conditions, whereas industrial settings are subject to environmental temperature fluctuations, reactor aging, and raw material inconsistencies. These factors introduce challenges in maintaining consistent CNT growth rates and fiber quality. To bridge this gap, we propose utilizing machine learning-driven adaptive control systems that dynamically adjust production parameters in real time. While progress has been made in research settings, translating these advancements to industrial-scale production will require further refinement. Future efforts should focus on refining ML-based optimization techniques and exploring alternative reactor configurations to enhance CNT fiber quality and production efficiency.

Action #3: The following text has been improved to the revised manuscript:

In section 2.1: "However, while reducing turbulence and controlling temperature gradients have been effective in small-scale experiments, maintaining these parameters in large-scale FCCVD reactors remains a major challenge. The complex gas dynamics in industrial reactors can lead to inconsistent CNT growth, negatively impacting fiber uniformity and mechanical performance. To address this, integrating computational fluid dynamics (CFD) simulations and real-time process monitoring into reactor design is essential to ensure stable production conditions. These strategies help optimize flow dynamics, reduce turbulence-induced inconsistencies, and improve CNT fiber uniformity at an industrial scale."

In section 2.2: "Although multi-component carbon sources have been shown to enhance CNT growth, their industrial application must also consider factors such as cost, stability, and byproduct generation. Similarly, while MOF-derived catalysts demonstrate high efficiency, concerns persist regarding their large-scale synthesis and long-term stability. Future research should focus on developing cost-effective, high-stability catalysts and optimizing continuous catalyst feeding strategies to maintain consistent CNT growth in industrial production."

In section 2.3: "Laboratory studies typically optimize individual variables under controlled conditions, whereas industrial production must account for environmental temperature fluctuations, reactor aging, and raw material inconsistencies. These factors introduce significant challenges in maintaining consistent CNT growth rates and fiber quality. To bridge this gap, machine learning-driven adaptive control systems can dynamically adjust production parameters in response to real-time process data. While significant progress has been made in research settings, translating these advancements to industrial-scale production requires further refinement. Future efforts should refine ML-based optimization techniques and

explore alternative reactor configurations to enhance CNT fiber quality and production rates."

Comment #4. *Machine learning (ML) is briefly mentioned as a tool for optimizing CNT fibers production. A deeper discussion is anticipated to explain the process of adjusting reaction conditions by optimizing model parameters dynamically. Furthermore, a comparison of ML-based approaches with traditional methods, particularly in terms of efficiency improvements, would strengthen the manuscript.*

Response #4: Thanks for your thoughtful and constructive suggestion. Machine learning (ML) requires a substantial amount of high-quality data for effective model training, while the floating catalyst chemical vapor deposition (FCCVD) process is complex, time-consuming, and challenging to operate. Consequently, only two published studies have explored the application of ML in CNT fiber production using the FCCVD method. Most ML models and algorithms aimed at optimizing CNT growth are based on the array growth process, which shares some similarities with FCCVD. Despite these challenges, ML has demonstrated its ability to significantly enhance experimental efficiency and accelerate research timelines. To provide a more detailed discussion, we have expanded our analysis of ML models and their potential applications in CNT fiber production. Additionally, we have addressed the economic feasibility of scaling up production by discussing how ML-driven optimization can reduce costs and improve process efficiency.

Action #4: The following text has been improved to the revised manuscript:

(*Paragraph 3, 4* on *Page 11* in the revised manuscript Chapter 2.3)

"Due to the numerous variables involved in the CNT production process, traditional trial-and-error experimental methods are insufficient for optimizing parameters. Recent studies have introduced machine learning (ML) techniques to navigate the multidimensional parameter space, identify optimal synthesis conditions, and facilitate the development of CNTs with specific properties. Multiparameter optimization models establish correlations between synthesis parameters (e.g., carbon feed, temperature, and catalyst amount) and CNT properties, enabling predictive control over fiber quality, yield, and scalability. However, the application of ML in FCCVD-based CNT synthesis remains limited. Most ML models are designed for the CVD method used in CNT array growth, which shares some similarities with FCCVD. ML algorithms—including artificial neural networks (ANN), random forests (RF), support vector machines (SVM), eXtreme gradient boosting (XGBoost), and Bayesian

optimization (BO)—have demonstrated high accuracy in modeling CNT array growth.[38-41] Furthermore, ML has been shown to significantly improve experimental efficiency and shorten research timelines. For instance, dynamic optimization via BO accelerated experimental speed by more than five times.[42] When combined with automation, over 500 experiments and millions of virtual simulations were completed within just 43 days, achieving the highest precision in producing HACNT arrays at a specified density.[43] From an economic perspective, integrating ML into industrial-scale CNT production offers promising advantages. ML-driven optimization can help unlock the complex growth mechanisms in FCCVD, bridge the performance gap between experimental and theoretical values, and reduce production costs and time compared to traditional methods. These advancements are expected to accelerate the industrialization of CNT fibers, making scalable manufacturing more economically viable.

Comment #5. *This manuscript addresses challenges and potential solutions for CNT fibers at macroscale levels. A more detailed analysis focusing on the synergistic and antagonistic effects of these proposed methods is expected to offer valuable insights.*

Response #5: Thanks for your valuable feedback. CNT fibers at the macroscale are primarily modified through three methods: mechanical processing, heat treatment, and modification treatments. These techniques enhance the mechanical and electrical properties of CNT fibers through different mechanisms. The properties can be further improved by combining these methods in a synergistic way. However, in the post-treatment process, introducing second phases may disrupt the inherent excellent properties of CNT fibers. Therefore, various factors must be carefully balanced during production. And we have revised the manuscript to address these points.

Action #5: The following text has been improved to the revised manuscript:

(*On Page 18* in the revised manuscript Chapter 3.3)

"Mechanical processing primarily enhances the high-density stacking of CNTs through physical methods, while modification treatments strengthen inter-tube connections via chemical approaches. Heat treatment improves the structural order by repairing defects. These techniques collectively enhance the mechanical and electrical properties of CNT fibers. During modification and heat treatment, additional substances or atoms may be introduced, adjusting fiber properties through mechanisms such as interfacial engineering and energy

band modulation. While these secondary phases can be beneficial, they may also disrupt CNT alignment and hinder the expression of the fibers' intrinsic properties. Therefore, multiple factors affecting fiber properties must be carefully balanced during post-treatment."

Comment #6. *It would be beneficial to consult more recent work about high performance fibers and their applications. The works on high performance fibers (SusMat. 2024, 4, e205; eScience, 2024, 4, 100250; Adv Mater., 2024, 2407009; Adv. Fiber Mater. 2022, 4, 1572–1583.) could be used as reference.*

Response #6: Thanks for your valuable suggestion. The works have been cited as ref. [72-74] in manuscript:

(*Paragraph 3* on *Page 21* in the revised manuscript Chapter 5)

"The future development of fiber materials is moving towards intelligence and multifunction.[72-74] With high-conductive and high-strength, the advanced applications of CNT fibers are vast, such as flexible wearable devices, energy storage, biomedical engineering, and aerospace."

Technical Responses to Referee #3

Comment #0. *This review has shown the recent progress and potential challenges in CNT fiber research from production to application, providing valuable guidance and insights for the CNT fiber research community. I would like to recommend the acceptance of paper after a minor revision.*

Response #0: Thank you for evaluating our manuscript and we greatly appreciate your valuable suggestions. Below, we provide point-by-point responses to address your questions and comments, and we have incorporated the suggested changes into the revised manuscript. The main revisions are highlighted in blue for clarity.

Comment #1. *Regarding the floating CVD based continuous growth of CNTs, although the authors have presented some progress in dynamic reaction simulations, catalyst design, carbon source optimization, and growth mechanism etc., it would be beneficial to add some recent breakthroughs in catalyst designs and growth rate control in continuous synthesis processes.*

Response #1: Thank you for your valuable suggestions. We have expanded the discussion to include recent breakthroughs in catalyst design and growth rate control for continuous CNT synthesis. Specifically, we have added a section focusing on the development of advanced bimetallic catalysts and nano-engineered supports, which enable more precise control over catalyst particle size and distribution. These innovations significantly enhance both growth rates and uniformity. Additionally, we have revised our section on metal-organic frameworks (MOFs), highlighting their ability to provide a consistent supply of high-surface-area cobalt nanoparticles. This contributes to enhanced growth kinetics and improved structural uniformity of CNTs.

Action #1: The following text has been improved to the revised manuscript:

(*On Page 9* in the revised manuscript Chapter 2.2)

"Recent researches in catalyst design, exemplified by advanced bimetallic systems and nano-engineered supports integrated into continuous synthesis processes, have enabled unique control over catalyst particle size and distribution, thereby markedly accelerating CNT growth

rates and enhancing uniformity.[31] Thus, the choice of catalyst precursors plays a crucial role in tuning the reaction dynamics and optimizing CNT synthesis."

(*On Page 9* in the revised manuscript Chapter 2.2)

"Moreover, recent optimizations using metal–organic frameworks (MOFs) such as ZIF-67 have further refined continuous synthesis processes by ensuring a steady supply of high-surface-area cobalt nanoparticles, thus bolstering both growth kinetics and structural uniformity of the CNTs."

Comment #2. *Concerning the CNT fiber property enhancement, the authors addressed the importance of the cross-scale assembly challenges, it's better to make a comparative analysis of different interface engineering routes (e.g., covalent functionalization vs. π-π interactions) and their respective strengthening efficiencies, providing instructive interfacial regulating strategies toward creative applications.*

Response #2: Thanks for your kind comments and suggestions. Since the effect of different interface engineering strategies on properties is strongly determined by the structure and material properties, we cannot specifically calculate the respective strengthening efficiencies. Here, we provide a comparative description of the mechanisms and characteristics of the interface engineering strategies. The following table has been added to the revised manuscript.

Action #2: The Table 4 is presented below:

(*Table 4* on *Page 17* in the revised manuscript Chapter 3.3)

Table 4. The comparison of various interface engineering methods

**Method**	**Mechanism**	**Advantages**	**Challenges**
Covalent functionalization	Covalent bonds (e.g., C-O, C-N)[67]	Strong bonding, stable	CNT damage, complex process
π-π interactions	Van der Waals forces (π-clouds)[68]	No CNT damage, simple	Weak binding, low stability
Interfacial enhanced phase	Stress dispersion in multi-phase composites[69]	Multifunctionalizable	Poor compatibility, non-uniform
Hierarchical interfaces	Load transfer optimization[66, 67]	Resilient, designable	Complex fabrication

oc-2025-00155p.R2

Name: Peer Review Information for "Advancing Carbon Nanotube Fibers: Addressing Challenges from Production to Application"

Second Round of Reviewer Comments

Reviewer: 1

Comments to the Author

Review Report to Authors

The manuscript presents a well-structured and in-depth overview of the multiscale challenges in carbon nanotube (CNT) fiber production, particularly focusing on the floating catalyst chemical vapor deposition (FCCVD) method, and potential solutions leveraging reactor design, catalyst optimization, and machine learning. While the article is commendable in its technical rigor and breadth of topics, several specific areas require further refinement to ensure a more comprehensive and impactful contribution.

Major Comments and Required Revisions

1. Economic and Environmental Impacts of Large-Scale Production

- Issue: While the article provides a solid technical discussion of production bottlenecks, it omits a rigorous analysis of economic feasibility and environmental sustainability—two critical factors for industrial viability.
- Required Revision: Include a subsection (possibly within Section 4 or as a separate final discussion point) that evaluates:
    - o Estimated production costs (materials, energy, maintenance).
    - o Life cycle environmental impacts (e.g., carbon footprint of FCCVD vs. wet spinning).
    - o Potential of biomass carbon sources to reduce environmental burden (as introduced on page 10–11).

o	Discussion of cost/benefit trade-offs between high-performance fibers and ecological considerations.

2. Clinical Translation in Biomedical Applications

•	Issue: The biomedical application section (page 21 onward) briefly notes the biocompatibility of CNTs but lacks depth on clinical translation pathways, including regulatory, safety, and in vivo performance issues.

•	Required Revision:

o	Discuss specific challenges in long-term in vivo performance (e.g., biodegradability, immune response).

o	Include examples or case studies of clinical trials or preclinical investigations of CNT-based fibers for neural, cardiovascular, or orthopedic uses.

o	Suggest pathways to enhance stability and biocompatibility (e.g., coating techniques, biofunctionalization).

3. Generalization of Optimization Strategies

•	Issue: Strategies like machine learning integration and reactor redesign (page 12–13) are promising, but the discussion generalizes their applicability across all FCCVD-based systems.

•	Required Revision:

o	Clarify that these approaches may not be universally applicable across different reactor scales and precursor systems.

o	Provide specific examples of when these techniques succeed or fail (e.g., high- vs. low-pressure systems, ferrocene vs. MOF-derived catalysts).

o	A decision tree or schematic for selecting suitable optimization approaches under varying conditions would be valuable.

4. Lack of Experimental Validation

•	Issue: The paper heavily references theoretical modeling, ML predictions, and literature reports without providing any experimental data or validation from the authors' lab.

•	Required Revision:

o If possible, include summary results or a supplementary data table from your own experiments (e.g., length/yield of CNT fibers under optimized conditions).

o Alternatively, critically evaluate existing experimental datasets and comment on reproducibility or gaps.

5. Narrow Range of Application Examples

• Issue: Applications are mostly limited to wearables, energy devices, biomedical sensors, and aerospace (page 20–22), missing industrial sectors like construction, filtration, defense, and robotics.

• Required Revision:

o Broaden Section 5 by adding:

 High-impact potential in structural reinforcement (e.g., CNT-reinforced cement or coatings).

 Fire-resistant or conductive textile applications.

 Smart robotic actuators and EMI shielding in harsh environments.

6. Insufficient Depth on Industrial Reactor Implementation

• Issue: Section 4.1 discusses reactor design and carbon deposition mitigation, but lacks real-world examples or data from pilot/industrial-scale setups.

• Required Revision:

o Cite or summarize industrial FCCVD reactor examples (e.g., pilot-scale setups from national labs or companies).

o Discuss challenges like maintenance cycles, reactor fouling, scalability limits, and control over multivariate parameters in real-time.

7. Development of Cost-Effective Catalysts

• Issue: The manuscript discusses MOFs and ferrocene derivatives (page 9–11) but lacks commentary on cost efficiency and recyclability.

• Required Revision:

o Discuss catalyst costs per gram and recyclability.

o Suggest strategies for catalyst recovery or reuse in FCCVD systems.

o Evaluate trade-offs between high activity and stability vs. cost and scalability.

8. Optimization of Catalyst Feeding

• Issue: Although continuous catalyst feeding is mentioned in Table 2 (page 13), its integration into automated production pipelines is not discussed.

• Required Revision:

o Expand on technical setups for catalyst injection, flow rate control, and challenges in maintaining steady-state operation.

o Include feedback control methods (e.g., ML-based real-time monitoring of catalyst activity) if available.

9. Innovation in Multiscale Performance Optimization

• Issue: Section 2.3 mentions machine learning (ML) but is focused more on lab-scale simulation.

• Required Revision:

o Provide a clearer roadmap for scaling ML tools from lab to industrial settings.

o Address integration with sensor feedback, digital twins, or automated process control.

o Explain the practical barriers to ML integration in legacy production environments.

10. Bioinspired Adhesives for Reinforcement

• Issue: The role of mussel-inspired adhesives (page 17–18) is briefly noted, but lacks material comparisons.

• Required Revision:

o Compare different bioinspired adhesives (e.g., tannic acid–gelatin blends, catechol-functionalized polymers) in terms of strength enhancement, thermal stability, and processing compatibility.

o Highlight potential for medical or marine applications.

11. Macroscopic Property Translation Limitations

• Issue: The paper discusses densification and alignment improvements (page 14–16), but glosses over remaining barriers like residual voids, interfacial slippage, or mechanical fatigue under repeated loading.

• Required Revision:

o Identify bottlenecks in fully translating intrinsic CNT properties into macroscale fibers.

o Include quantitative performance gaps and strategies for minimizing them (e.g., cyclic load fatigue tests, polymer anchoring techniques).

12. Scalable Fabrication Methodologies

• Issue: The need for scalable methods is acknowledged, but the review lacks comparative performance analysis of different methods beyond Table 1.

• Required Revision:

o Expand Table 1 or add a new comparative figure showing performance (e.g., strength, conductivity, cost, throughput) per method.

o Highlight trade-offs in detail.

Minor Suggestions

• Figure Captions: Ensure all figures have self-contained captions. Figure 2 and Figure 3 are detailed but may benefit from clearer legends for symbols and abbreviations.

• Table Formatting: Improve visual clarity of Table 2 and Table 4 (e.g., use color shading or icons for performance advantages).

• Language: Minor grammatical errors exist (e.g., “concomitantly” is overused, and plural agreement in several places should be reviewed).

Recommendation

Major Revision Required – The manuscript is well-structured and scientifically promising but requires a deeper integration of economic, clinical, and industrial considerations, as well as more critical analysis of generalizability, validation, and practical barriers.

Reviewer: 2

Comments to the Author

The authors have respond well to the questions, now this paper can be accepted.

Author's Response to Peer Review Comments:

Editor, ACS Central Science

Re: ACS Central Science manuscript oc-2025-00155p titled “Advancing Carbon Nanotube Fibers: Addressing Challenges from Production to Application” by Guo et al.

Dear Editor,

Thank you for your email and for the provisional acceptance of our manuscript titled "Advancing Carbon Nanotube Fibers: Addressing Challenges from Production to Application." We sincerely appreciate your support and the opportunity to move forward toward publication in ACS Central Science.

We have carefully reviewed the formatting requirements and will submit a clean version of the manuscript without highlights, colored text, or tracked changes. The marked-up version will be submitted as "Supporting Information for Review Only."

Please let us know if any further action is required. We look forward to your final decision.

Best regards

Huisheng PENG
